# Network hub gene detection using the entire solution path information

**DOI:** 10.1093/genetics/iyae187

**Published:** 2024-11-13

**Authors:** Markku Kuismin, Mikko J Sillanpää

**Affiliations:** Research Unit of Mathematical Sciences, University of Oulu, P.O. BOX 8000, Oulu FI-90014, Finland; Research Unit of Mathematical Sciences, University of Oulu, P.O. BOX 8000, Oulu FI-90014, Finland

**Keywords:** hub identification, high-dimensional data, penalization methods, gene co-expression networks, hub glasso, network learning

## Abstract

Gene co-expression networks typically comprise modules and their associated hub genes, which are regulating numerous downstream interactions within the network. Methods for hub screening, as well as data-driven estimation of hub co-expression networks using graphical models, can serve as useful tools for identifying these hubs. Graphical model-based penalization methods typically have one or multiple regularization terms, each of which encourages some favorable characteristics (e.g. sparsity, hubs, and power-law) to the estimated complex gene network. It is common practice to find a single optimal graphical model corresponding to a specific value of the regularization parameter(s). However, instead of doing this, one could aggregate information across several graphical models, all of which depend on the same data set, along the solution path in the hub gene detection process. We propose a novel method for detecting hub genes that utilizes the information available in the solution path. Our procedure is related to stability selection, but we replace resampling with a simple statistic. This procedure amalgamates information from each node of the data-driven graphical models into a single influence statistic, similar to Cook’s distance. We call this statistic the Mean Degree Squared Distance (MDSD). Our simulation and empirical studies demonstrate that the MDSD statistic maintains a good balance between false positive and true positive hubs. An R package MDSD is publicly available on GitHub under the General Public License https://github.com/markkukuismin/MDSD.

## Introduction

Reverse engineering of gene co-expression networks has emerged as a valuable tool for exploring gene interactions in genetics. Unlike in classical graph theory, where the relationships encoded in the network are clearly defined, the construction of gene networks is not a straightforward task. In this work, the terms graph and network are used interchangeably. A prime example of a network in classical graph theory is the renowned “Paul Erdős collaboration network,” which maps the extensive collaborations of the prolific mathematician Paul Erdős (Newman 2001). In this collaboration network, the connections are unambiguous and free of any kind of estimation error. When investigating a set of genes, it is not atypical to lack prior biological information from other sources for integration into the graphical model (unsupervised setting), but instead having only indirect observations like gene expression levels. As a result, the edges of gene co-expression networks are not univocal, affected by both measurement and estimation errors (Wang and Huang 2014; van Dam *et al.* 2018).

Typically, the edges represent either pairwise linear relationships between genes (Pearson’s correlation coefficient) or pairwise linear relationships with the effect of confounding variables (genes) removed (partial correlation coefficient). Estimates of these coefficients must be derived from the data. As a result, a certain level of uncertainty is always inherent in data-driven network learning. Another complicating factor in the construction of data-driven networks is that graphs are usually high-dimensional, implying that the number of genes may substantially exceed the number of data samples (see, e.g. Meinshausen and Bühlmann 2006). It is not uncommon for the number of genes to be tens of thousands of times greater than the number of available samples (see, e.g. Verhaak *et al.* 2010).

After constructing the gene co-expression network, one can examine its structure to gain insights not only about pairwise relationships between genes, but also about how genes interact as a group. Genes with similar co-expression patterns are examined to detect gene modules, which might be phenotype-specific. Moreover, such gene modules often contain hub genes. Hub genes, which are connected to several other genes within the module, may play an essential role in the functionality of the gene module and the gene network as a whole (Barabasi and Oltvai 2004; Hao *et al.* 2012; Langfelder *et al.* 2013; van Dam *et al.* 2018; Liu *et al.* 2020).

We assume that it is possible to gain more insight into the hub genes not by inspecting just one graphical model, but by extracting information jointly from several data-driven networks, all of which depend on the same data. It involves changing the value of the penalty term in these methods to observe how the relationships between genes change. In particular, changing the value of the penalty term alters the degree of a gene (node), that is, the number of genes directly connected to it. These different penalized models form what is known as the network solution path.

We propose using a hub screening method, which is similar to stability selection (Meinshausen and Bühlmann 2010) using a statistic somewhat similar to Cook’s distance (Cook 1977). Specifically, our goal is to utilize the entire solution path to gather more information in high-dimensional cases. Our measure aggregates over the solution path of each gene with respect to the estimated degree of gene, by comparing a gene with other genes. This procedure is loosely related to SIgnificant ZERo crossings of derivatives (SiZer) (Chaudhuri and Marron 1999, 2000) which aims to utilize the information available over different smoothing levels. Similarly, our purpose is not to try to find the true underlying gene network, but rather to view the true underlying gene network at different tuning parameter levels. See also Holmström and Pasanen (2017), Lockhart *et al.* (2014), and Kuismin *et al.* (2020).

The hub screening measure described here is applicable to any probabilistic graphical model [e.g. the Gaussian graphical model (GGM), the covariance graph model, the binary Ising model, and dynamic network models] and any network estimation method using a user-specified tuning parameter(s). For simplicity, we focus on the GGM and estimators suitable for the construction of GGM (Kuismin and Sillanpää 2017; Qiao *et al.* 2018). Specifically, we pay attention to the $L_{1}$ penalization methods, as these can be used to estimate the partial correlation between genes. This helps us to understand direct relationships between genes, excluding the influence of other genes, which can be referred to as confounding genes. This can be particularly useful in complex gene networks where many genes are interrelated. Examples of $L_{1}$ penalization methods include the Graphical least absolute shrinkage and selection operator (glasso) (Banerjee *et al.* 2008; Friedman *et al.* 2008), Sparse partial correlation estimation (space) (Peng *et al.* 2009), constrained $L_{1}$-minimization for inverse matrix estimation (CLIME) (Cai *et al.* 2011), the hub graphical lasso (Tan *et al.* 2014; Wang *et al.* 2023), scale-free graphical lasso (Liu and Ihler 2011), and degree weighted lasso (DWLasso) (Sulaimanov *et al.* 2018), among others. See Wang and Huang (2014), Drton and Maathuis (2017) and Qiao *et al.* (2018) for a more extensive survey in the literature.

## Materials and methods

The general framework for any nondynamic, undirected graphical model can be summarized as follows: Consider a multivariate random vector $Y=(Y_{1},Y_{2},\ldots ,Y_{p}{)}^{⊤}$ that contains information on gene expressions on genes. The expression levels of the genes *i* and *j* are denoted with two random variables, $Y_{i}$ and $Y_{j}$, respectively, i,j=1,…,p. An undirected graph *G* consists of two finite sets, G=(V,E), where V={i|i=1,…,p} is a finite set of nodes (genes) and E⊆{(i,j)|i,j∈V,i≠j} is a set of edges that correspond to gene-by-gene co-expression relationships. The graphical model G encodes the pairwise undirected co-expression relationship between all genes i=1,…,p. In the case of GGM, we assume that the random vector Y follows the multivariate normal distribution N(0,Σ), where 0 is a *p*-dimensional zero vector and $\Sigma =[\sigma_{ij}]$ is a symmetric, p×p positive definite covariance matrix. The set of edges E can be constructed using the nonzero elements of the precision matrix $\Theta =\Sigma^{-1}=[\theta_{ij}]$ as follows: (i,j)∈E if and only if $\theta_{ij}\neq 0$, i≠j. Under these assumptions, the edge between genes *i* and *j* denotes a significant partial correlation. In practice, the precision matrix (edge set E) is unknown and must be estimated from the data. Thus, the data-driven network depends on the estimated precision matrix $\hat{\Theta }={\hat{\Sigma }}^{-1}$. We can estimate the covariance matrix *Σ* using the sample covariance matrix *S*. However, when the number of samples *n* is smaller than the number of genes *p*, the sample covariance matrix *S* is not positive definite and is not invertible. Therefore, in the high-dimensional case, the GGM cannot be constructed from the data using either the maximum likelihood or the unbiased estimator of the sample covariance matrix.

A popular method for GGMs in high-dimensional data is the glasso estimator. Denote the negative log-likelihood of a multivariate normal variable Y with l(Θ,Y). Let *λ* denote a positive tuning parameter and $\|\cdot {\|}_{1}$ is the $L_{1}$-norm. The solution to the following minimization problem over nonnegative definite precision matrices provides the glasso estimate of the precision matrix *Θ*,


(1) $$\underset{\Theta \geq 0}{\arg\;\min}\;\{l(\Theta ,Y)+\lambda \|\Theta {\|}_{1}\}.\;$$

One of the beneficial properties of the glasso estimator is that it provides a positive definite estimate of the precision matrix, even in high-dimensional cases. Furthermore, the glasso estimator is sparse, which means that some off-diagonal elements of the estimated precision matrix are exactly zero. The number of these zero entries depends on the value of the tuning parameter. As the tuning parameter approaches zero, the precision matrix estimate becomes a full matrix. Conversely, as the tuning parameter increases, the estimate of the precision matrix becomes a diagonal matrix (Friedman *et al.* 2008).

The convention with GGMs is to first estimate several networks and then select the “best” graphical model for a more detailed inspection. For glasso, numerous model selection criteria are available, including the Bayesian Information Criterion (BIC), extended BIC (eBIC) (Chen and Chen 2008; Foygel and Drton 2010), Akaike’s Information Criterion (AIC), cross-validation (CV), Stability Approach for Regularization Selection (StARS) (Liu *et al.* 2010), and Rotation Information Criterion (RIC) (Lysen 2009; Zhao *et al.* 2012). Moreover, there are criteria specifically developed for complex graphical models with gene modules, such as path connectivity (PC), AGlommerative NESted (AGNES) (Mestres *et al.* 2018), and gap-com statistic (Kuismin *et al.* 2022), just to mention a few examples of which we are aware.

There are variants of the glasso estimator specifically designed for complex gene co-expression network learning with hub nodes. The space procedure (Peng *et al.* 2009) is a modification of the network neighborhood selection method (Meinshausen and Bühlmann 2006) that can control the overall sparsity of the partial correlation matrix. The authors argue that this approach is more powerful in identifying hubs than glasso. Liu and Ihler (2011) proposed an estimator that encourages the network to have a more scale-free structure. The hub graphical lasso (hglasso) (Tan *et al.* 2014; Wang *et al.* 2023) can be particularly useful for network hub detection and it can be used to detect hub genes of a very high degree. These “super-hub” genes can regulate numerous nonhub genes, resulting in denser connections. Thus, the hglasso method should be an effective approach for learning gene co-expression networks with super-hub genes (Tan *et al.* 2014). We note that these types of dense connections are not typical in a scale-free network; although scale-free networks are a very important class of complex networks in gene co-expression analysis (Zhang and Horvath 2005; Langfelder and Horvath 2008; Caron and Fox 2017), there are no clear hub nodes in a scale-free network.

The hglasso algorithm is based on the following decomposition of the precision matrix: $\Theta =V+V^{⊤}+Z$, where *Z* and *V* are sparse, symmetric p×p matrices. The columns of the matrix *V* correspond to the hub nodes of the network, and nonzero elements of *Z* correspond to edges between nonhub nodes. Denote the $L_{2}$-norm with $\|\cdot {\|}_{2}$. The solution to the following minimization problem provides the hglasso estimate of the precision matrix *Θ*,


(2) argminΘ≥0,V,Z{l(Θ,Y)+λ1‖Z−diag(Z)‖1+λ2‖V−diag(V)‖1∑j=1p+λ3∑j=1p‖V−diag(V)j‖2},subject toΘ=V+V⊤+Z.

In contrast to glasso, hglasso uses three penalty terms with corresponding tuning parameters to encourage more hub-like structure to the derived network. In particular, $\lambda_{1}\geq 0$ controls the sparsity of the matrix *Z*, $\lambda_{2}\geq 0$ controls the sparsity of hub nodes, and $\lambda_{3}\geq 0$ controls the selection of the hub nodes. The formalization of hglasso, proposed by Wang *et al.* (2023), extends hglasso to a scenario where one has prior information available about the hub nodes. This estimator includes five tuning parameters. In this paper, we only consider data-driven networks when we lack prior biological information from other sources.

To the best of our knowledge, there is no time-efficient model selection criterion available for hglasso that could be used to select the “best” values for each tuning parameter when constructing a high-dimensional graph. Tan *et al.* (2014) proposed using a variant of the BIC for model selection with hglasso. Their simulations show that this BIC-type quantity (hereafter BIC) favors graphical models with hubs.

In the next section, we introduce a novel screening method, similar to stability selection (Meinshausen and Bühlmann 2010), using a statistic somewhat similar to Cook’s distance (Cook 1977) which can be used to examine the hubs of a data-driven network. This method is applicable to any graph learning method in which the sparsity of the network is controlled by a tuning parameter or a set of tuning parameters. Our procedure can facilitate hub detection when the network is very large and when the degree of the hub nodes (super-hubs) is high, making the visualization of the entire network infeasible.

### Hub screening using mean degree squared distance

There is not a rigid mathematical definition for a hub node. The standard procedure for determining a hub node involves selecting a set of top nodes with the highest degrees compared to the degrees of other nodes in the network. These top ones are then designated as hub nodes. We see no drastic need to change the current standards, but we do propose some minor enhancements on how hubs are detected: (1) given that the number of hub genes is typically much smaller than the number of nonhub genes, the squared Euclidean pairwise distance between the degree of a hub gene and of any other gene in the network is large, (2) this difference is noticeable while inspecting the data-driven networks with respect to different tuning parameter values.

Our approach shares similarities with the stability selection (Meinshausen and Bühlmann 2010). Stability selection has been utilized earlier in graphical model selection to select a single optimal tuning parameter value (Liu *et al.* 2010). However, the data resampling step of the stability selection makes it time-intensive method in graphical model selection. Instead of data resampling, we want to propose a time-efficient hub screening method. Thus, rather than computing the solution path over several data subsamples, we propose a statistic which also provides a practically interesting graphical interpretation about the (hub) nodes of the data-driven network. Like outlier detection methods, such as Cook’s distance (Cook 1977), we compare how the node degree of a single node differs from all other nodes of the whole graphical model.

We propose the following statistic for hub detection that collects information over the network solution path. Denote Λ={λ|λ>0} a sequence of values of the tuning parameters in no particular order, |Λ|=m. Let ${\hat{d}}_{i}(\lambda )$ denote the estimated degree of node *i* depending on a value of the tuning parameter λ∈Λ, i=1,…,p. The Mean Degree Squared Distance (MDSD) of node *i* for an estimator using just one tuning parameter is determined as follows:


(3) $${\mathrm{MDSD}}_{i}=\frac{1}{m(p-1)}\sum_{\lambda \in \Lambda }\sum_{\begin{array}{c} \;j=1\\ j\neq i \end{array}}^{p}[{\hat{d}}_{i}(\lambda )-{\hat{d}}_{j}(\lambda ){]}^{2}.$$

**Fig. 1.**
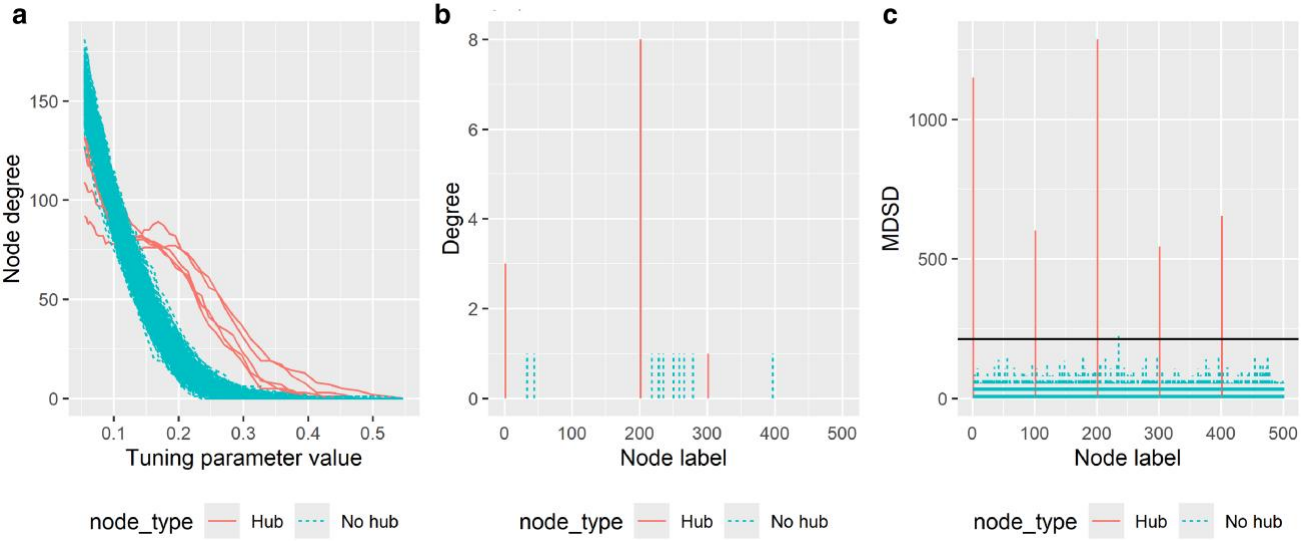
a) Glasso solution path of 500 nodes in simulated example data when n=110. Estimated node degrees are plotted against the tuning parameter values (here, |Λ|=50). b) Degree of the data-driven graphical model when RIC is used as a graphical model selection criterion. c) MDSD values for each node. The solid vertical line corresponds to the 3MDSD_p_ cutoff value. The true hub nodes (5 hubs) are illustrated with solid lines, and nonhub nodes with dashed lines. The graphical model considered here is a star graph.

In Fig. 1, it is evident that hub nodes with a significantly higher degree have correspondingly higher estimated node degree values relative to the value of the tuning parameter, compared to other nodes. Similarly, the MDSD values of hub nodes are seemingly greater than those of nonhub nodes. However, the node degree of an estimated graphical model alone is not always the best indicator for hub nodes as shown in Fig. 1b. In this figure, we have illustrated the estimated node degrees in case when RIC (Zhao *et al.* 2012) is used as a graphical model selection criterion. In this example, only two hub nodes are found. Moreover, when the sample size is very small, conventional model selection criteria tend to select very sparse, even empty graphical models (results not shown). On the other hand, MDSD is likely to facilitate hub detection by emphasizing larger differences between the degrees of hub nodes compared to nodes with a low degree. Additionally, MDSD can provide extra graphical information when working with small samples.

The MDSD can be generalized to accommodate approaches that utilize multiple tuning parameters. Denote $\Lambda_{1}$, $\Lambda_{2}$, …, $\Lambda_{q}$ set of different tuning parameters with no specific order such that $|\Lambda_{k}|=m_{k}$, k=1,…,q. Denote $M=\prod_{k=1}^{q}m_{k}$. Let ${\hat{d}}_{i}(\lambda_{1},\ldots ,\lambda_{q})$ denote the estimated degree of node *i* depending on tuning parameter values $\lambda_{1}\in \Lambda_{1},\ldots ,\lambda_{q}\in \Lambda_{q}$. The general form of the MDSD statistics is as follows:


(4) $$\begin{array}{rl} {\mathrm{MDSD}}_{i} & =\frac{1}{M(p-1)}\sum_{\lambda_{1}\in \Lambda_{1}}\cdots \sum_{\lambda_{q}\in \Lambda_{q}}\sum_{\begin{array}{c} \;j=1\\ j\neq i \end{array}}^{p}[{\hat{d}}_{i}(\lambda_{1},\ldots ,\lambda_{q})-{\hat{d}}_{j}(\lambda_{1},\ldots ,\lambda_{q}){]}^{2}. \end{array}$$

To address the question of which nodes should be declared as hub nodes relative to other nodes in the network, we propose the following decision rule that can serve as a general guideline for detecting significant hub nodes. That is, a node *i* is classified as a significant hub node (hub gene) if the corresponding value of ${\mathrm{MDSD}}_{i}$ exceeds a given threshold value, i.e. ${\mathrm{MDSD}}_{i}>\gamma {\mathrm{MDSD}}_{p}$, where ${\mathrm{MDSD}}_{p}=(1/p)\sum_{i=1}^{p}{\mathrm{MDSD}}_{i}$, and *γ* is a positive constant, i=1,…,p. We propose using either the 2.5 or 3 for *γ* here (see different recommendations for outlier detection; Rousseeuw and Hubert 2011). The example of using cutoff value $3{\mathrm{MDSD}}_{p}$ to declare hub nodes is shown in Fig. 1. Note that any cutoff value should not be applied without careful consideration. We examined how the choice of the threshold value *γ* affect the False Discovery Rate (FDR) of the hub detection using simulated data. These results show that when an adequate graphical model estimator is used, the FDR is low with a wide range of threshold values *γ* (see Supplementary Section 2, Figs. S21–S26).

Given that MDSD is a metric akin to Cook’s distance, the value of ${\mathrm{MDSD}}_{i}$ could also be high when the estimated degree of node *i* is low and there are numerous high-degree nodes in the network. However, this is contrary to what we observe in complex gene networks in reality. Nevertheless, we cannot dismiss the possibility of encountering such results when applying MDSD in hub detection with high-dimensional data. These nodes can be easily identified by plotting both the node degree with respect to the values of the tuning parameters and the MDSD values as illustrated in Fig. 1. In such instances, it is crucial to carefully verify whether the estimator and the corresponding tuning parameter values are inappropriate for the network learning task, potentially resulting in drastically dense network models.

**Fig. 2.**
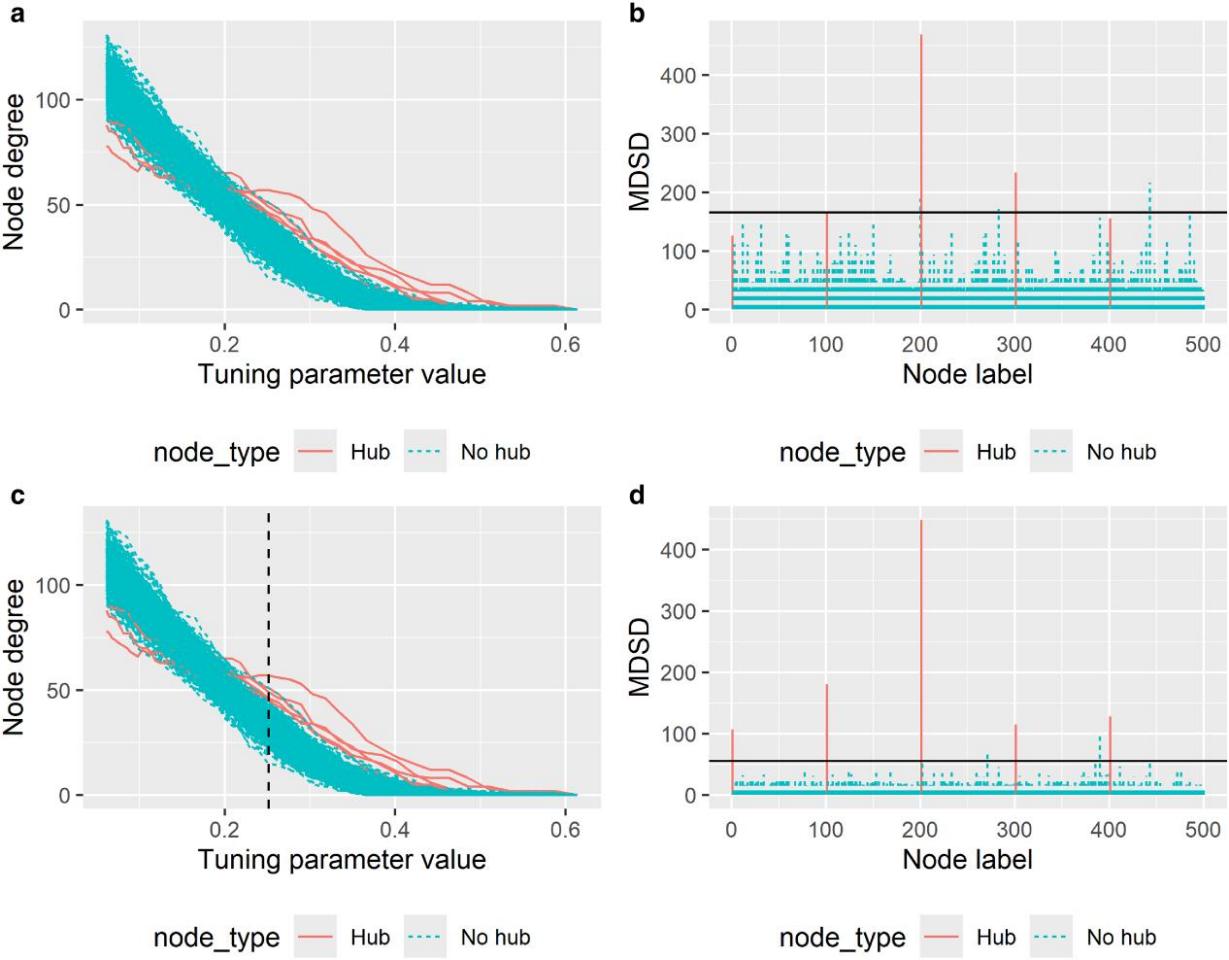
a) Glasso solution path of 500 nodes in simulated example data when n=50. Estimated node degrees are plotted against the tuning parameter values (here, |Λ|=50). b) MDSD values for each node. The solid vertical line corresponds to the ${3\mathrm{MDSD}}_{p}$ cutoff value. c) Estimated node degrees are plotted against the tuning parameter values. The vertical dashed line illustrates the largest tuning parameter value which returns a model with estimated degree distribution skewness smaller than 0.5. d) MDSD values for each node when models with the estimated degree distribution skewness smaller than 0.5 are ignored when computing MDSD. The solid vertical line corresponds to the ${3\mathrm{MDSD}}_{p}$ cutoff value when the skewness cutoff is applied. The true hub nodes (5 hubs) are illustrated with solid lines, and nonhub nodes with dashed lines. The graphical model considered here is a star graph.

Considering that data-driven graphical models do not always resemble realistic gene networks with a power-law degree distribution (Barabási and Albert 1999), we could obtain more informative MDSD values by ignoring part of the solution path information (all implausible graphical models). Thus, we propose that before plotting the MDSD statistic, one could check that each of the included node degree distributions follows a realistic co-expression network that is, which approximately follows the power-law. In particular, skewness can be used as a criterion, as power-law distribution is positively skewed (say, degree distribution with skewness >0.5). We have illustrated this behavior in Fig. 2 where MDSD values are first calculated over all data-driven network models, and then MDSD is calculated only for subsets of networks showing degree distribution which are positively skewed. As one can see in Fig. 2b, when all models are considered, it is difficult to distinguish hub nodes from other nodes using MDSD. On the other hand, when the skewness of the estimated degree distribution is considered, the MDSD values of the true hub nodes are much pronounced compared to other nodes (Fig. 2d).

**Fig. 3.**
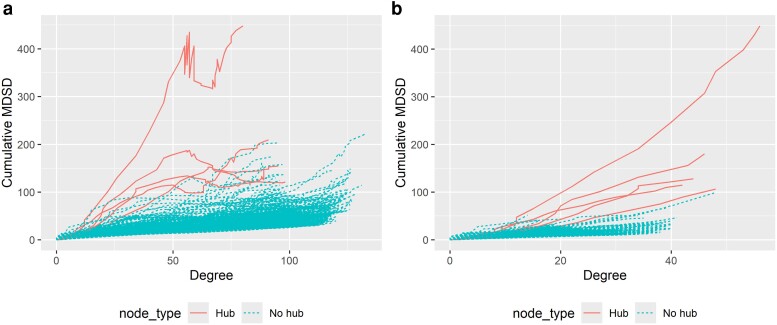
a) Glasso solution path of 500 nodes in simulated example data when n=50. The cumulative MDSD values are plotted against the estimated node degrees. b) The cumulative MDSD values are plotted against the estimated node degrees after removing such models from the solution path whose estimated degree distribution skewness is smaller than 0.5. The true hub nodes (5 hubs) are illustrated with solid lines, and nonhub nodes with dashed lines. The graphical model considered here is a star graph.

In Fig. 3, we have presented the cumulative MDSD values as a function of estimated node degrees. As one can see from Fig. 3a, the cumulative MDSD is not a monotonic function of the node degree. In practice, we have noticed that the degree distributions of the graphical models computed with small tuning parameter values do not follow a power-law distribution (see Fig. 2c). When graphical models whose degree distributions do not resemble a power-law distribution are removed, hub nodes can be more easily separated from low-degree nodes. Consequently, the true hub nodes logically yield the largest MDSD values.

## Simulation examples

### Methods

Are the MDSD values really drastically higher for the hub nodes compared to the other nodes? Moreover, could the use of the entire solution path information improve the detection of hub nodes? The purpose of these numerical examples is to illustrate that MDSD really yields reasonable candidates for potential hub genes. In addition, we demonstrate that the use of MDSD can improve hub detection, compared to the “common” procedure. In this study, we employ hglasso to learn a sparse GGMs. We adopt the BIC-type quantity proposed by (Tan *et al.* 2014) as our baseline model selection criterion (common procedure). This BIC-type quantity utilizes the hglasso decomposition of the precision matrix, and particularly the hub nodes found with hglasso. To our knowledge, it is the only model selection tool specifically aimed at detecting hubs with hglasso. Using other graphical model selection tools with hglasso (e.g. eBIC) is somewhat problematic, because they do not utilize the hub information which we get from the matrix decomposition. We tested this: if the eBIC is used directly to the precision matrix estimated with hglasso ignoring the matrix decomposition, the results are clearly inferior compared to the BIC-type quantity proposed by Tan *et al.* (2014) (results not shown). Subsequently, we calculate the MDSD for each node derived from the hglasso solution path and calculate the MDSD only for a subset of networks showing the degree distribution with skewness >1. A node *i* is classified as a hub node if ${\mathrm{MDSD}}_{i}>{3\mathrm{MDSD}}_{p}$. hglasso has three tuning parameters and we select these in the same way as in the simulation example in Tan *et al.* (2014). We fix $\lambda_{1}=0.4$, consider three values of $\lambda_{3}$ (0.5, 1, and 2), and use a grid with 10 values, evenly spaced between 0.1 and 1 for $\lambda_{2}$. We utilize hglasso R package when computing the respective hglasso estimates.

In addition to hglasso, we use two other estimators with MDSD to detect hub nodes: (1) a fast approximation of the partial correlation graph using the so-called lossy screening rule (thresholding), that is, if the absolute value of the Pearson correlation coefficient between two variables *i* and *j* is greater than a threshold value *λ*, that is, $|r_{ij}|>\lambda$, where λ∈[0,1], then there is an edge between nodes *i* and *j*; otherwise, there is no (Fan and Lv 2008; Zhao *et al.* 2012), and (2) the space method (Peng *et al.* 2009). We utilize the space R package when computing the space estimates. For comparison, we also use another quick hub screening method presented in Hero and Rajaratnam (2012), Firouzi and Hero (2013) (shortly FH screening). While using the space methods, we apply a BIC-type criterion proposed by Peng *et al.* (2009). Unlike hglasso, space does not provide any tools to identify which nodes are hub nodes. Therefore, when we use BIC while selecting the network derived with space, we rank the nodes by their degree and select the top $h_{n}$ nodes with the highest degree as hub nodes. In this context, $h_{n}$ is equal to the true number of network hubs, as determined from the true graphical model. See Supplementary Materials for these additional examples.

### Simulation models

We consider five graphical models, which we use to generate 100 replicated data sets from a multivariate Gaussian distribution.

1. Hub-network. This network contains so-called super-hubs (Tan *et al.* 2014; Wang *et al.* 2023).
2. Two component hub-network. This network is composed of two super-hub networks (Tan *et al.* 2014; Wang *et al.* 2023).
3. Star network with different numbers of stars (Zhao *et al.* 2012).
4. The scale-free network (Barabási–Albert) (Barabási and Albert 1999).
5. Inter-hub network. This network has both intramodular hub nodes and an intermodular hub. The intermodular hub node is not a hub in the sense that its degree is quite low compared to intramodular hubs, but it works as an important bottleneck node in a network (van Dam *et al.* 2018; Liu *et al.* 2020).

In these simulation examples, the sample size and the number of variables, denoted (n,p), are (100,500) or (500,1,500).

Clear hub nodes exist in the hub-network, the two-component hub-network and the star network, which have a higher total degree compared to other nodes. The nodes considered hubs in the scale-free network do not have a distinctly high node degree compared to other nodes in the network. However, the scale-free network has network characteristics that are common in a variety of complex gene networks (Langfelder *et al.* 2013; van Dam *et al.* 2018; Liu *et al.* 2020). We use the procedures from the R packages igraph (Csárdi *et al.* 2024), hglasso, and huge (Zhao *et al.* 2012) during our data simulation. All graphical models are described in detail and visualized in the (see Supplementary Figs. S1–S5).

### Performance metrics

To determine how well hubs detected with MDSD compare to hubs identified by BIC-type criteria or FH screening, we calculate six metrics as a measure of the quality of hub detection: the False Detection Rate (FDR), False Positive Rate (FPR), Precision (Pre), and True Positive Rate (TPR or sensitivity),

-
$\text{FDR}=\frac{\mathrm{FP}}{\mathrm{FP}\;+\;\mathrm{TP}}$ .
-
$\text{FPR}=\frac{\mathrm{FP}}{\mathrm{FP}\;+\;\mathrm{TN}}$ .
-
$\text{Pre}=\frac{\mathrm{TP}}{\mathrm{TP}\;+\;\mathrm{FP}}$ .
-
$\text{TPR}=\frac{\mathrm{TP}}{\mathrm{TP}\;+\;\mathrm{FN}}$ .

where TP is the number of true positives, FP is the number of false positives, TN is the number of true negatives, and FN is the number of false negatives. In our simulations, a “true positive” finding corresponds to a correctly identified hub node, while a “false positive” corresponds to an incorrectly identified hub node. Similarly, a “true negative” finding corresponds to a correctly identified nonhub node, and a “false negative” corresponds to an incorrectly identified nonhub node.

A high Pre but low TPR indicates that the detected hub nodes are indeed hub nodes, but the statistical power of the hub detection method is low. Conversely, a low Pre but high TPR suggests that many correct hub nodes are detected, but among the detected hub nodes, there are many nodes which are not hubs in reality. Therefore, we use the Matthews Correlation Coefficient (MCC) and Bookmaker Informedness (BM) to evaluate the overall hub detection performance,

-
$\mathrm{MCC}=\frac{\mathrm{TP}\times \mathrm{TN}-\mathrm{FP}\times \mathrm{FN}}{\sqrt{\left(\mathrm{TP}+\mathrm{FP})\times (\mathrm{TP}+\mathrm{FN})\times (\mathrm{TN}+\mathrm{FP})\times (\mathrm{TN}+\mathrm{FN}\right)}}.$

-
BM=TPR−FPR .

**Fig. 4.**
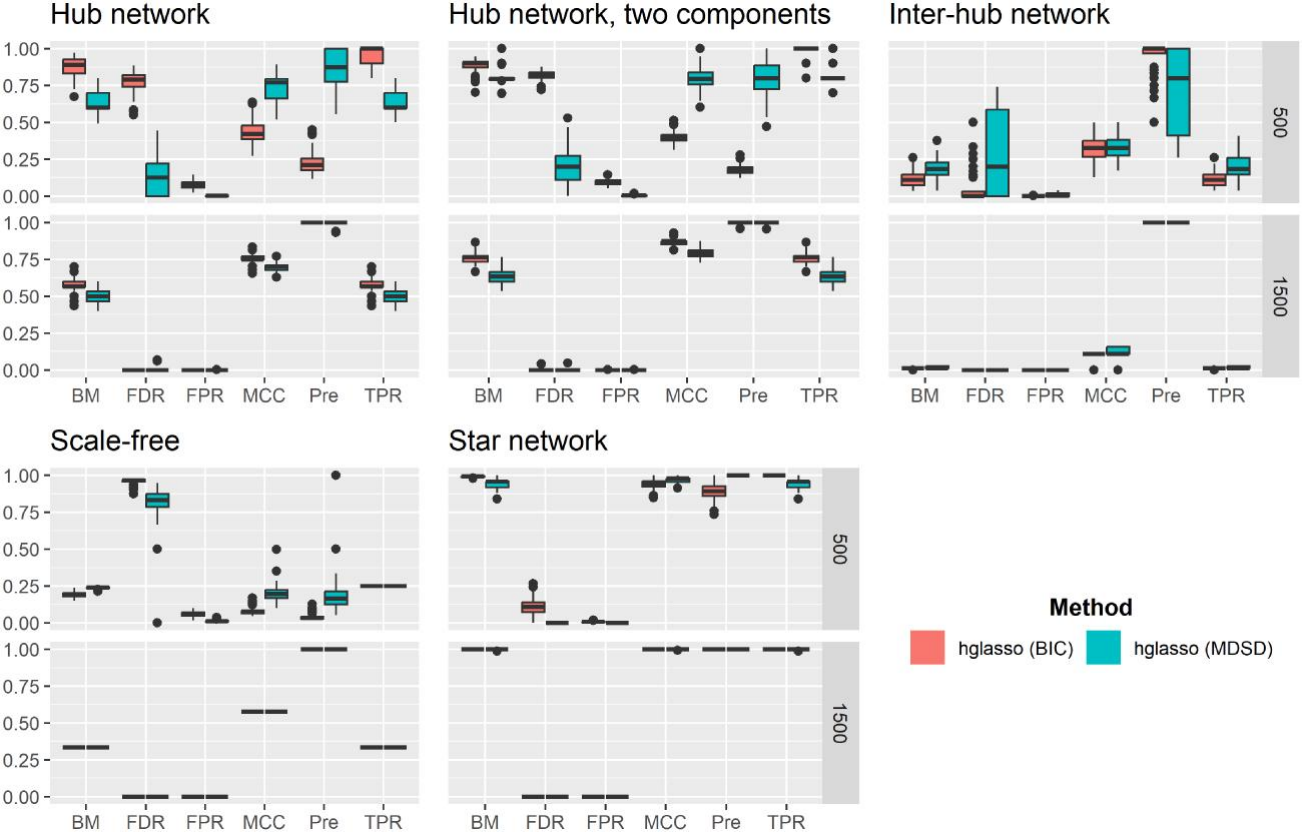
Averaged values of binary classification metrics, estimated from 100 simulation replications, are presented when hglasso is used to detect hubs with either BIC-type quantity (BIC) or MDSD. Hub detection methods are distinguished using different colors and grouped boxplots. A high-quality version of the results can be found in the Supplementary Materials.

**Fig. 5.**
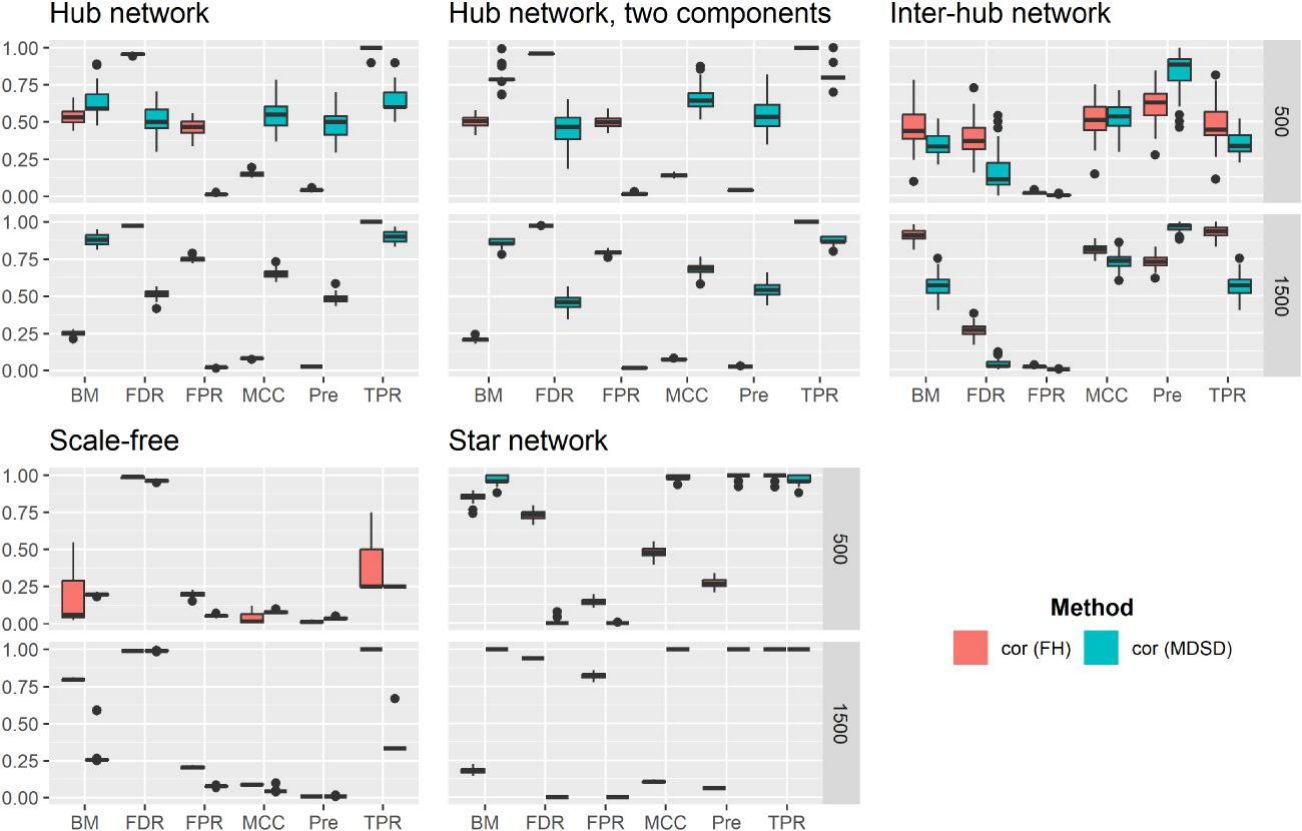
Averaged values of binary classification metrics, estimated from 100 simulation replications, are presented when correlation thresholding (cor) is used to detect hubs with either FH screening or MDSD. Hub detection methods are distinguished using different colors and grouped boxplots. A high-quality version of the results can be found in the Supplementary Materials.

**Fig. 6.**
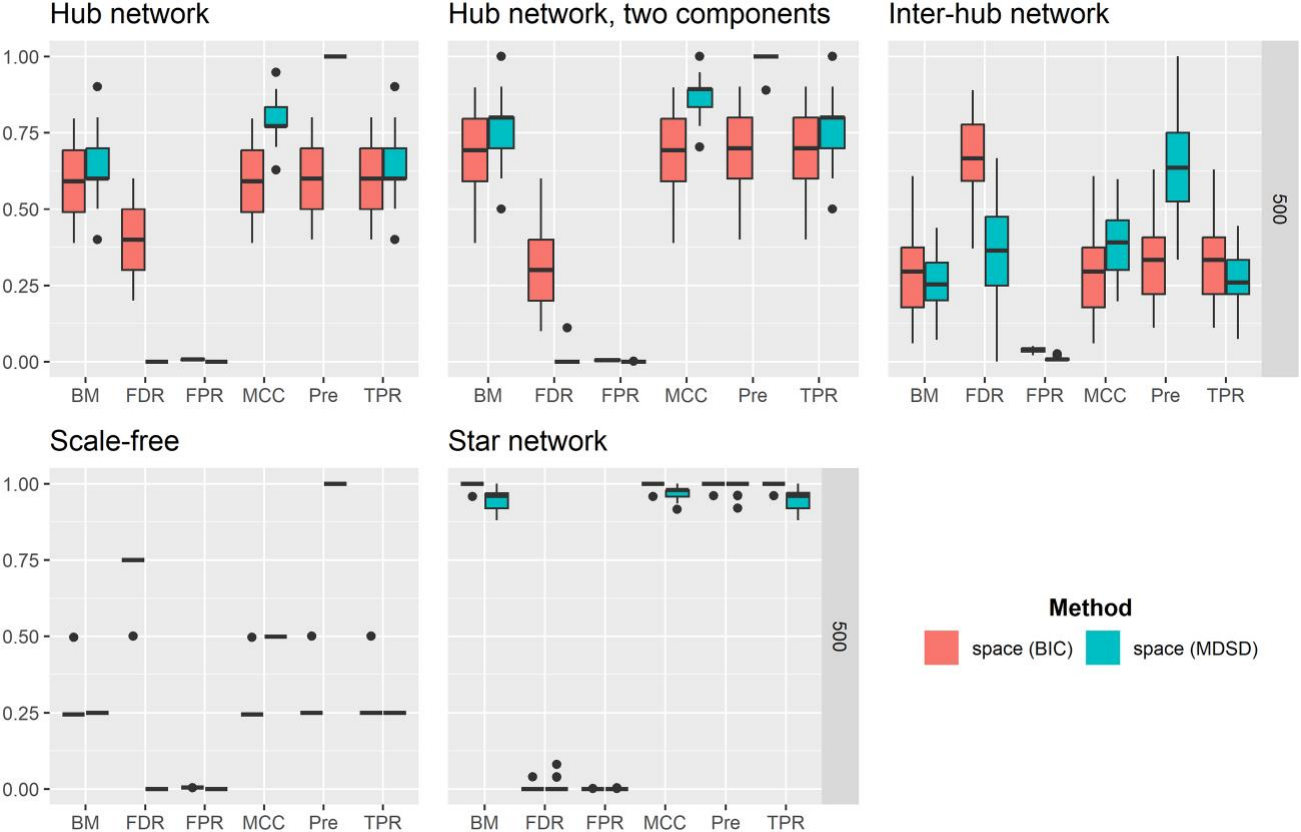
Averaged values of binary classification metrics, estimated from 100 simulation replications, are presented when space is used to detect hubs with either BIC or MDSD. Hub detection methods are distinguished using different colors and grouped boxplots. A high-quality version of the results can be found in the Supplementary Materials.

Both MCC and BM can vary between −1 and 1, assuming that no denominator in MCC equals zero. A BM or MCC value close to one indicates that the classification method has a good overall performance. Because there is no consensus on the most informative statistic to evaluate the general quality of a binary classification task (Zhu 2020; Chicco, Tötsch, *et al.* 2021; Chicco, Warrens, *et al.* 2021), we report both the MCC and BM values, taking into account that the number of nonhub nodes far exceeds the number of hub nodes (imbalanced dataset). The average performance metrics for hglasso, correlation thresholding, and space are summarized in Figs. 4–6, respectively. High-quality versions of these figures are reported in the Supplementary Materials. Moreover, we used the Mann–Whitney *U*-test to compare different performance metrics between different methods. *P*-values of these test are reported in (see Supplementary Figs. S6–S20).

### Simulation results

It is remarkable that when using the MDSD cutoff value ${3\mathrm{MDSD}}_{p}$ with hglasso, the averaged MCC and Pre values of the MDSD hub detection procedure are substantially higher compared to the graphical model selected with BIC. This occurs when p=500, with the exception of the inter-hub network model (P-value<0.01). This is not surprising because hglasso is not intended for this type of specialized network setting (Tan *et al.* 2014).

Higher TPR values are associated with higher averaged BM values. A more detailed inspection of the results reveals that the corresponding number of FPs is quite high (results not shown). It is known that the BIC tends to favor dense graphical models (Drton and Maathuis 2017). However, the number of FPs decreases as the number of variables and the number of samples increases, and the difference between the averaged values of BM and MCC associated with BIC and MDSD decreases. In the case of hub-network and two component hub-network, BIC is associated with higher average BM values (P-values<0.01). This is probably due to the fact that the same set of tuning parameter values was used while inspecting all graphical models. It is possible that hglasso could perform better with different tuning parameter values, but detailed hyperparameter tuning is not feasible in this kind of simulation study. However, these simulations show that MDSD can be used to distinguish the hub nodes from other nodes more efficiently than BIC while using hglasso.

When considering the scale-free graphical model, the average values of both BM and MCC are higher when MDSD is used with space compared to the BIC-type criterion of Peng *et al.* (2009) (P-value<0.01). In addition, the averaged value of Pre is much higher when MDSD is used (P-value<0.01). The authors argued that space should work well while learning scale-free networks. This example shows that MDSD improves the overall performance of the estimator, assuming that the estimator itself is well suited for the graphical model learning task (see Fig. 6 and Supplementary Fig. S19).

If the network estimation method is not well-suited for the task of learning the network model in the first place, this inadequacy will also be reflected in subsequent MDSD values. For example, MDSD did not perform as well as the hub screening method of Firouzi and Hero (2013) when inspecting the inter-hub network or the scale-free network with the correlation thresholding. Looking at the averaged BM and MCC values suggests that the correlation thresholding does not produce a network with a scale-free degree distribution. This is quite evident when examining the results for the scale-free network models when P=1,500; the average values of both MCC and BM are very modest and the average FDR values are extremely high with both FH screening and MDSD (see also Supplementary Figs. S14 and S15). When we apply either hglasso or the space method to these models, the average binary classification metrics improve substantially. This is the case even though the BIC-type criterion performs better with the space method when considering the star network (MCC and BM P-values<0.01; see Fig. 4 and Supplementary Fig. S20). However, when estimating super-hub networks with distinct hub nodes using correlation thresholding or space methods, the averaged BM and MCC values are far higher when using MDSD in hub detection compared to FH screening and BIC (P-value<0.01; see Supplementary Figs. S11–S12 and S16–S17).

Detecting hubs within the inter-hub network model proved to be a challenging task for all penalization methods considered in this simulation study. Surprisingly, the simple correlation thresholding (lossy screening rule) was actually the best strategy to find both inter- and intramodular hubs. This outcome can be explained by the fact that the degree of the node is not a sufficient statistic to explore intermodular hub(s). Instead of node degree, the important nodes of the network with possible intermodular hubs could be summarized more comprehensively using the centrality of betweenness (van Dam *et al.* 2018). However, the $L_{1}$ penalization methods and the FH screening method only use the degree of the node while learning undirected graphical models. In addition to Figs. 4–6, see also Supplementary Figs. S8, S13, and S18.

## Real data examples

We examine expression levels of two real data sets, plant and human, using hglasso. In particular, we examine gene expression of *maize ligule* on *Zea mays* (Johnston *et al.* 2014) and of human breast cancer (Xie *et al.* 2017). We aim to derive a gene co-expression network to detect hub genes. Such hub genes likely play an important role during plant development and disease pathway by regulating many other downstream genes. We find that the data-driven co-expression network constructed from *Zea mays* data shows clear hub-like structure, whereas the hubs in the network constructed from the breast cancer data are much less pronounced. Nevertheless, the prediction model constructed from these hub genes indicate that these hub gene candidates have higher predictive-ability compared to other genes and therefore are important players in the gene co-expression network.

We use the hglasso implemented in the R package hglasso to construct a network with hubs and MDSD to examine potential hub genes using the information over the entire hglasso solution path. To investigate these potential hub genes in more detail, we examine how each hub gene is able to discriminate (i) *lg1* mutant samples in the *maize ligule* data, (ii) tumor and healthy samples in the breast cancer data from other samples using logistic regression. We conducted a receiver operating characteristic curve (ROC) analysis and calculated the gene-specific area under the curve (AUC) (Fawcett 2006). We use the R package pROC to perform our analysis (Robin *et al.* 2011).

### *Maize ligule* hub nodes

We examine the expression levels of genes related to the *Maize ligule*. Overall, there are expression levels for 109,715 genes across 24 samples. In particular, among these samples, 3 are *liguleless1 (lg1)* mutants. Compared to other samples, the *lg1* mutants lack both ligules and auricles, and their leaves are narrower and more upright than their wild-type siblings (Johnston *et al.* 2014). The expression data are publicly available at The National Center for Biotechnology Information (NCBI) Gene Expression Omnibus under accession number GSE61333.

After normalizing the expressions and using a variance stabilizing transformation to make the expression values approximately homoskedastic using the R package DESeq2 (Love *et al.* 2014), we select the 1,000 genes with the highest variance to ease the graphical model construction process and the reproducibility of these examples: It takes about 5 h to construct the network graphical models with the tuning parameter values we have used (R version 4.3.1 running on a laptop, 64-bit operating system, 32 GB RAM, 2.70 GHz processor).

**Fig. 7.**
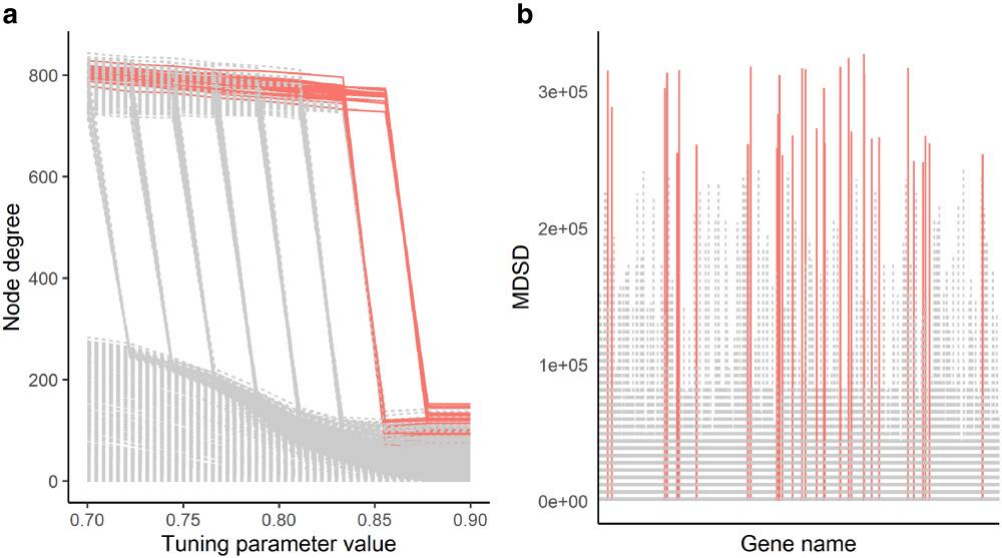
a) The hglasso solution path of 1000 differentially expressed genes (nodes). Estimated node degrees are plotted against the tuning parameter values (in this case, $\lambda_{2}$). b) MDSD values for each gene. The genes with the MDSD values greater than the threshold ${3\mathrm{MDSD}}_{p}$ are illustrated with solid red lines in both panels. Names of the potential hub genes are reported in Table 1.

We follow the same practice as in Tan *et al.* (2014) when selecting the three tuning parameters for hglasso. First, we fix $\lambda_{1}=0.9$ so that the matrix *Z* is sparse, $\lambda_{3}=20$ to obtain only a few potential hub genes, and values ranging from 0.7 to 0.9 for the parameter $\lambda_{2}$. Then we apply hglasso with this set of tuning parameters to the empirical correlation matrix. For comparison, we also selected the graphical model using the BIC-type quantity (hereafter BIC). We use the cutoff value γ=3 when detecting the hub nodes. Node degrees associated with the hglasso solution path and MDSD values for each node are illustrated in Fig. 7. The hub genes detected with the BIC and MDSD are listed in Table 1.

**Table 1.** Putative hub genes identified using hglasso with BIC-type quantity and MDSD: we find 45 hub genes with hglasso together with the BIC and 32 potential hubs using MDSD ($\mathrm{MDSD}>{3\mathrm{MDSD}}_{p}$).

Prefix	Gene name
GRMZM#	**2G005818**, **2G007953**, **2G039934**, **2G040890**
	**2G047954**, **2G049681**, **2G060079**, **2G094639**
	**2G096372**, **2G112894**, **2G113349**, **2G114998**
	**2G116079**, **2G123308**, **2G128421**, **2G129413**
	**2G136364**, **2G140739**, **2G141955**, **2G151319**
	**2G155340**, **2G158281**, **2G167824**, **2G168002**
	**2G171852**, **2G176141**, **2G365134**, **2G377217**
	**2G402417**, **2G409372**, **2G416625**, **5G861547**
	2G030123, 2G068639, 2G074124, 2G092008
	2G092590, 2G094353, 2G100318, 2G119246
	2G119499, 2G162453, 2G358386, 5G804575
	5G859954

Common hub genes are boldfaced.

**Fig. 8.**
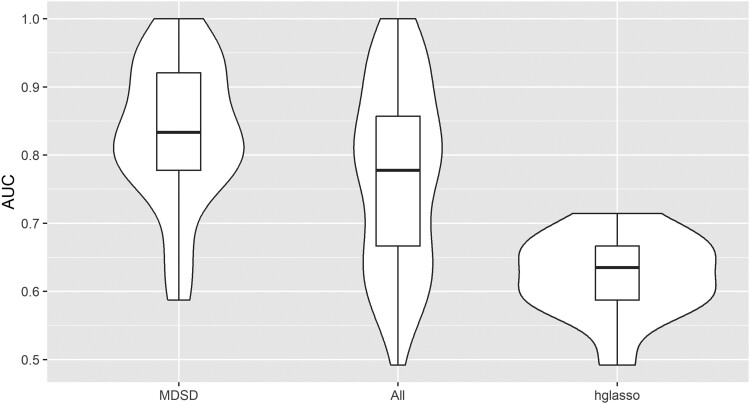
Averaged AUC values for each putative hub gene. The area around boxplots correspond to kernel probability density of AUC values (violin plot). (i) a subset of 32 hub genes which were detected with MDSD (MDSD larger than ${3\mathrm{MDSD}}_{p}$), (ii) 45 hub genes which were detected with hglasso, and (iii) a subset of 13 hub genes which were detected with hglasso but their corresponding MDSD values were lower than the threshold ${3\mathrm{MDSD}}_{p}$.

In this example, we find 32 hub genes using MDSD and 45 hub genes using BIC with hglasso (32 common hub genes). Of course, there are always false positives among these hub genes, even without acknowledging the extremely small sample size. Based on the simulation results of the two-component hub network, where the estimated FPR was slightly higher with the BIC-type criterion, we assume that there are fewer false positives among the hub genes detected with MDSD than among the hub genes detected with BIC. Further, we examine how each hub gene is able to discriminate *lg1* mutant samples from other samples using logistic regression. The AUC values averaged for all hub genes detected with hglasso, as well as hub genes unique for MDSD and hglasso are illustrated in Fig. 8 (compare to Table 1).

The hub genes detected with MDSD (MDSD larger than ${3\mathrm{MDSD}}_{p}$) are associated with higher AUC values compared to all other genes while discriminating *lg1* mutants from the other samples. In particular, putative hub genes, which were found only with hglasso (MDSD smaller than ${3\mathrm{MDSD}}_{p}$) are associated with much lower AUC values. This supports our assumption that some of the hub genes detected with hglasso can be considered false positives. The expression levels of the hub genes found using MDSD show better predictive performance when inspecting the development of maize *lg1* mutants.

### Breast cancer hub nodes

We examine the predictive ability of hub genes in a human disease-based dataset, focusing on the expression levels of genes related to breast cancer. Overall, there are 17,814 genes and 590 samples: 61 normal tissue samples and 529 breast cancer tissue samples. The expression data are publicly available at the Mendeley Data repository (Xie *et al.* 2017). See also Xie *et al.* (2016). In this case, we apply the fused graphical lasso (FGL) (Danaher *et al.* 2014) to jointly derive two gene co-expression networks simultaneously for both normal tissue genes and breast cancer tissue genes. Our idea is to borrow strength across these two classes to estimate two graphical models such that the final data-driven FGL networks will have shared edges between the healthy and breast cancer networks, as well as edges that are unique to each network. We will examine the shared and unique hubs of the normal and breast cancer tissue networks. First, we imputed missing co-expression values once. Because the proportion of missing values is <0.1% overall, we use simple mean imputation. After the imputation, we select the 1,000 genes with the highest variance to ease the graphical model construction process and to remove genes with variance attributable to nonbiological noise.

**Fig. 9.**
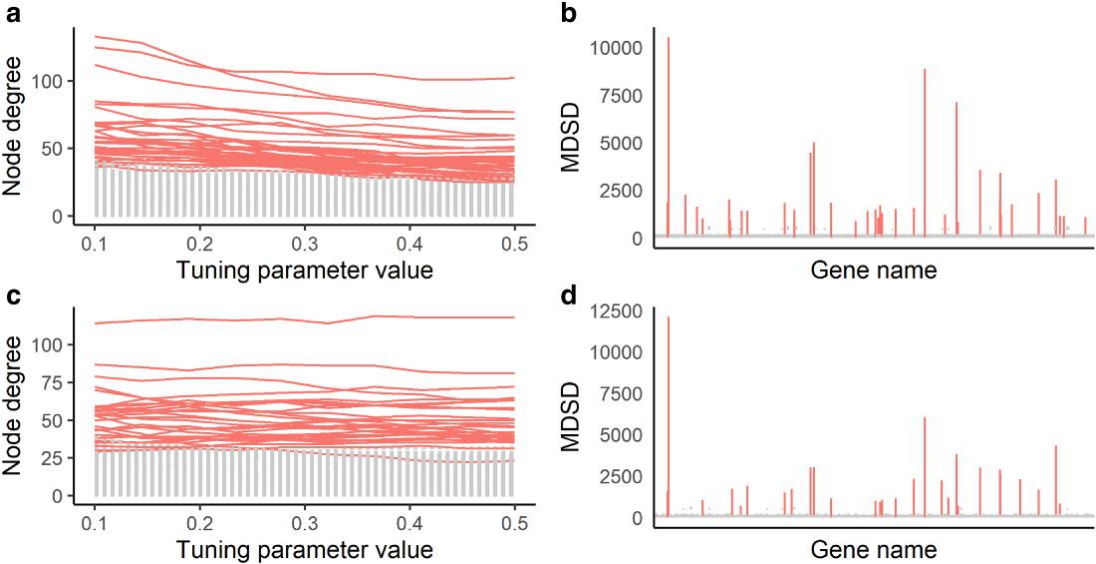
a) The FGL solution path of 1,000 differentially expressed genes (nodes) of the normal samples. Estimated node degrees are plotted against the tuning parameter values (in this case, $\lambda_{2}$). b) MDSD values for each gene of the normal samples. c) The FGL solution path of 1,000 differentially expressed genes (nodes) of the breast cancer samples. Estimated node degrees are plotted against the tuning parameter values (in this case, $\lambda_{2}$). d) MDSD values for each gene of the breast cancer samples. The genes with the MDSD values greater than the threshold ${3\mathrm{MDSD}}_{p}$ are illustrated with solid red lines in all panels. Names of the potential hub genes are reported in Table 2.

We follow the same practice as in the *Maize ligule* example when selecting the two tuning parameters for FGL. After examining the distribution of the sample correlation values, We fix $\lambda_{1}=0.9$ to ensure the precision matrices are sparse, and use values ranging from 0.1 to 0.5 for the parameter $\lambda_{2}$ to identify the edges that differ most strongly between normal and cancer networks. Then we apply FGL with this set of tuning parameters to derive breast cancer and normal tissue sample-specific networks. We use the cutoff value γ=3 when detecting the hub nodes using MDSD. Danaher *et al.* (2014) proposed to select the graphical model using an approximation of the AIC. However, unlike hglasso, this AIC approximation does not provide any tools for hub node/gene selection. Node degrees for both normal and breast cancer network solution paths, categorized by node and class, are illustrated in Fig. 9. The hub genes detected with MDSD are listed in Table 2.

**Table 2.** Putative hub genes identified using FGL with MDSD (MDSD greater than ${3\mathrm{MDSD}}_{p}$) in both normal and breast cancer class.

Normal tissue	Cancer tissue	Common
AGR2	AGR2	AGR2
AGR3	AGR3	AGR3
ATAD4	C1orf64	C1orf64
C10orf81	CEACAM6	COL17A1
C1orf64	CLCA2	EDN3
CDH1	COL17A1	FOXA1
CDH3	EDN3	G0S2
CLDN8	ESR1	GRP
COL17A1	FOXA1	KLK8
EDN3	G0S2	KRT23
FABP7	GRP	KRT6B
FOXA1	KLK8	LPL
G0S2	KRT23	MIA
GRP	KRT6B	MUCL1
IL6	LPL	PCOLCE2
KIAA1324	MIA	PDZK1
KLK8	MUCL1	PROM1
KRT15	NPY1R	ROPN1
KRT23	OGN	ROPN1B
KRT6B	PCOLCE2	SOSTDC1
LPL	PDZK1	TFAP2B
MIA	PROM1	TIMP4
MUCL1	ROPN1	
NTF5	ROPN1B	
PCOLCE2	SFRP1	
PDZK1	SOSTDC1	
PROM1	TFAP2B	
ROPN1	TIMP4	
ROPN1B		
RP13-102H20.1		
SCGB2A1		
SOSTDC1		
TFAP2B		
TIMP4		
TMPRSS2		
VTCN1		

Genes which are determined as hubs in both classes are reported in the rightmost column.

**Fig. 10.**
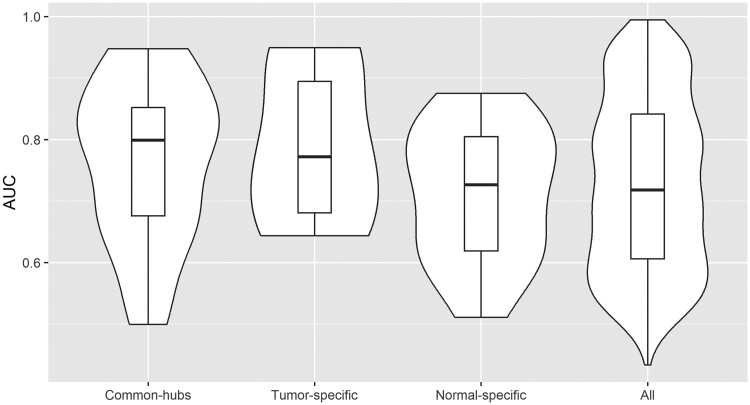
Averaged AUC values for each putative hub gene one at a time. The area around boxplots correspond to kernel probability density of AUC values (violin plot). (i) a subset of 22 hub genes found in both classes, (ii) 28 hub genes which were detected in the breast cancer class, (iii) 36 hub genes which were detected in the normal class, and (iv) all 1,000 genes. In both classes, a gene was determined as hub if the corresponding MDSD value was larger than ${3\mathrm{MDSD}}_{p}$.

In this example, we find 36 (28) hub genes using MDSD with FGL in the normal (breast cancer) class, respectively. Altogether there are 22 common hub genes, 6 hub genes unique in the breast cancer class, and 14 hub genes unique in the normal class. We examine how each of these unique hub gene can discriminate breast cancer tissue samples from normal tissue samples using logistic regression. For this analysis, we divide the data into a training and test data is such a way that we use approximately 70% of the samples to train logistic regression model. The rest of the samples are used to test the accuracy of the fitted model. The AUC values averaged for all hub genes detected using FGL together with MDSD are illustrated in Fig. 10 (compare with Table 2).

Although the discrimination accuracy of individual tumor-specific hub genes ($\mathrm{MDSD}>{3\mathrm{MDSD}}_{p}$) is not significant compared to the discrimination accuracy of all genes (P-value>0.1) when discriminating breast cancer tissue samples from normal tissue samples, these six (6) tumor-specific hub genes are associated with high AUC values compared to all other genes. These results indicate that MDSD together with FGL can be an effective tool for identifying a small subset of interesting gene co-regulations worth of further investigation.

**Fig. 11.**
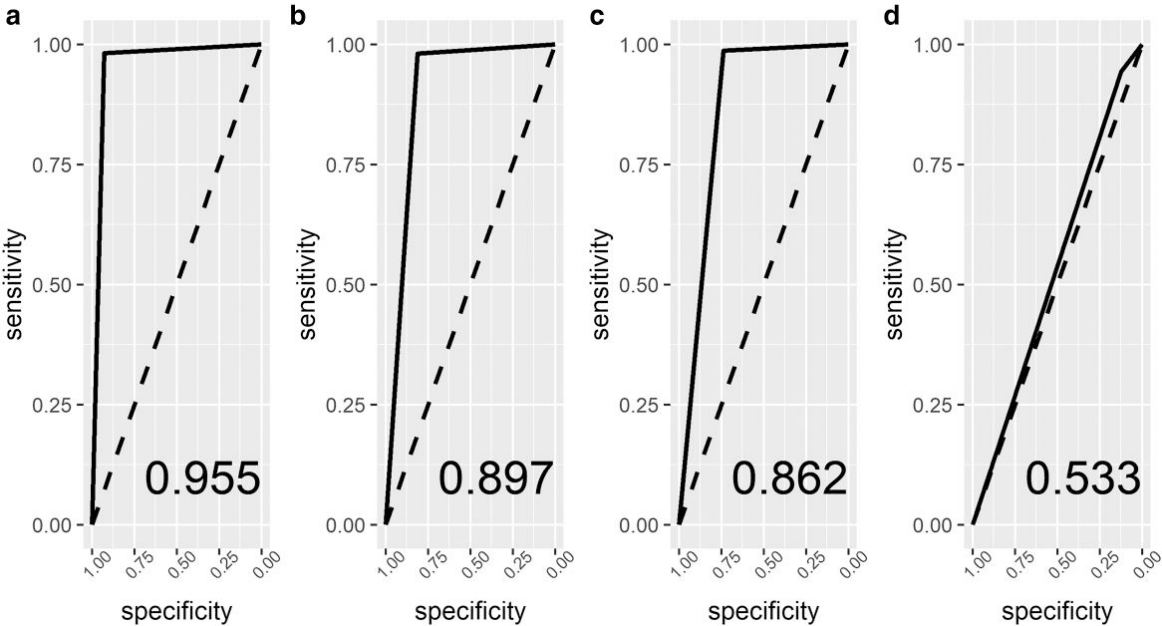
ROC curves of logistic regression models which is used to discriminate breast cancer tissue samples from normal tissue samples, when a) all tumor class-specific hub nodes are used as predictors, b) all normal class-specific hub nodes are used as predictors, c) both the tumor and normal hub nodes are used as predictors, and d) all genes in the data set are used as predictors. The diagonal dashed line corresponds to a random guess classification. The AUCs of the test data are reported in each panel.

We also examined the prediction accuracy of the logistic regression model when the potential hub nodes are used simultaneously as predictors. When all hub genes found unique only in the breast cancer samples network are used as predictors to discriminate normal samples from breast cancer samples, the corresponding AUC on the test set is approximately 0.955 and the corresponding 95% confidence interval is [0.884;1] indicating that the tumor-specific hub genes detected with MDSD play together an important role while trying to understand the development of breast cancer. The corresponding test classification accuracy for hub genes found only in the normal sample network is 0.897 and the corresponding 95% confidence interval is [0.798;0.996]. When hub genes from the both classes are used as predictors, the accuracy of the test classification is 0.862 and the corresponding 95% confidence interval is [0.760;0.964]. These results show that tumor sample-specific hub genes might have an important role in trying to understand the development of breast cancer. ROC curves for both groups are illustrated in Fig. 11. The discrimination methods reported in Xie *et al.* (2016) show similar test data classification accuracies with this same gene co-expression data set.

We finally note, that if we solely want to use detected hub genes in phenotype prediction, Kwon *et al.* (2023) have recently proposed a weighted stability selection approach which could be used together with MDSD presented here to find a set of hub genes with optimized predictive ability. In any case, our examples show that hub genes detected using MDSD are worth exploring in more detail.

## Discussion

Our simulation examples and real data analysis show that the information over the entire solution path can be utilized in hub detection. This information can even enhance the efficiency of an estimator in hub detection, assuming one employs the appropriate network estimator. The primary computational challenge in using MDSD for hub detection is the need to initially compute data-driven networks with certain discrete values of the tuning parameter(s). Path algorithms, such as Least Angle Regression (LARS), can be employed with the Lasso regression method to compute the entire solution path for all possible values of the tuning parameter in a time-efficient manner (Efron *et al.* 2004; Hoefling 2010; Tibshirani and Taylor 2011). Unfortunately, to the best of our knowledge, no such algorithm exists for GGM construction methods.

We would like to remind you that both MDSD and FH screening methods (Hero and Rajaratnam 2012; Firouzi and Hero 2013) are, more or less, graphical hub evaluation methods. When using the FH screening method, the waterfall plots provide more information about the potential hub nodes, rather than computing the *P*-values with fixed parameter values (Hero and Rajaratnam 2012). Similarly, MDSD values are also providing more information for hub detection in addition of node degree.

The penalty functions of the penalization methods $L_{1}$ depend on the degree of an individual node (1) or the degree of the hub node (2) (see also Liu and Ihler 2011). However, gene regulatory networks can contain both intermodular and intramodular hub genes. These types of hub nodes cannot be summarized solely by node degree. Although one can monitor the betweenness centrality using the entire information of the solution path, the potential of the information gained from this approach cannot be fully utilized due to the absence of learning methods that consider gene centrality in a gene regulatory network.

Although our method can avoid selecting a single optimal tuning parameter value, it is not robust for inadequate choice of graphical model (which is not supporting presence of hubs). For optimal hub gene detection accuracy, one should check that the estimated degree solution path is monotonically decreasing with increasing tuning parameter values. However, the solution paths of methods such as glasso are not always monotonic. Although we can somehow control this by picking a set of feasible tuning parameter values for glasso and other penalized graphical model estimators, handpicking a set of sufficient tuning parameter values is not a feasible solution while examining high-dimensional graphical models. However, we can improve our hub detection procedure by ignoring models from the solution path whose empirical degree distribution does not resemble a power-law distribution typical for hub networks.

Moreover, all these problems can be mitigated by using an adequate estimator for data-driven network construction. In the true data examples (*Maize ligule* and breast cancer), both solution paths are monotonic. In the breast cancer example, we find very informative hub nodes which could be used to generate biological hypotheses, for example, about the development of breast cancer.

## Supplementary Material

iyae187_Supplementary_Data

## Data Availability

*Maize ligule* expression data of Johnston *et al.* (2014) are publicly available at NCBI Gene Expression Omnibus under accession number GSE61333 https://www.ncbi.nlm.nih.gov/geo/query/acc.cgi?acc=GSE61333). The Cancer Genome Atlas Program (TCGA) gene expression profiles of breast cancer (Xie *et al.* 2017) are publicly available at Mendeley Data under CC BY 4.0 licence https://data.mendeley.com/datasets/v3cc2p38hb/1). The source codes used to produce the results and analyses are publicly available on a Github repository at https://github.com/markkukuismin/MDSD_supplementary. Supplemental material available at GENETICS online.
