# Supplementary material for: Network hub gene detection using the entire solution path information
Source: Genetics. 2024 Nov 13;229(1):iyae187. doi: 10.1093/genetics/iyae187 (PMC11708912; doi:10.1093/genetics/iyae187)
Supplement: iyae187_Supplementary_Data [file iyae187_supplementary_data.pdf]

Network hub gene detection using the entire solution path information - Supplementary materials

Markku Kuismin[1] and Mikko J. Sillanpää*[1]

[1]*Research Unit of Mathematical Sciences, University of Oulu, Finland*

1 Simulation examples

1.1 Simulation models

Denote $\Sigma^{-1} = \Theta = [\theta_{ij}]$ the symmetric and positive definite precision matrix which is the inverse of the covariance matrix Σ. We simulate the $n \times p$ data matrix Y from a multivariate normal distribution $N(0, \Sigma)$. Variables are standardized to have standard deviation one.

The zero and non-zero entries of Θ depend on the adjacency matrix of the corresponding graphical model. The adjacency matrix $A = [a_{ij}]$ is defined as follows: $(i, j) \in \mathbf{E}$ if and only if $a_{ij} = 1$, $i \neq j$, $i, j = 1, \ldots, p$, $a_{ii} = 0$. Thus, the non-zero and zero entries of Θ and A are equal while considering Gaussian Graphical Models (GGM).

Here we consider five (5) different graphical models:

1. Hub-network: This is a super-hub network. For all off diagonal elements of a symmetric adjacency matrix $A = [a_{ij}]$, we set $a_{ij} = 1$ with probability 0.01, and zero otherwise. The elements of the rows and columns of A corresponding to randomly selected hub nodes are set equal to one with probability 0.9 and zero otherwise.

2. Two component hub-network: This is a network with two connected components and super-hub nodes. The adjacency matrix is generated as $A = diag(A_1, A_2)$. Adjacency matrices A_1 and A_2 are generated following the hub-network scenario described above.

3. Star network: In a star network, non-nodes are connected to exactly one central hub node. In our model, we randomly designate $\lceil p/20 \rceil$ of the nodes as central hub nodes.

4. Scale-free network: This network is also known as the Barabási–Albert network. Here we use the stochastic algorithm to generate the scale-free graph. Specifically, the algorithm starts with a single node and no edges in the first time step. Then, one node is added in each step, and the new node initiates some edges to old nodes. The probability that an old node is chosen is given by $P(i) = d_i^{\alpha} + a$, where d_i is the degree of node i in the current step. In our simulation, we set $\alpha = 1.5$ and $a = 1$. We consider a model with at least three nodes that

*To whom correspondence should be addressed: mikko.sillanpaa@oulu.fi

have a degree greater than or equal to $0.05(p-1)$. These nodes are then considered as hub nodes.

5. Inter-hub network: This network consists of several hub nodes and one node that connects all these hub nodes together. Initially, a Star graph is generated following the procedure described above for the Star graph. Then, we add one inter-modular hub node to the graph. This hub node serves to connect all the intra-modular hubs together.

The precision matrices are determined given the sparse adjacency matrices corresponding to each graphical model. The super-hub network models and their corresponding data matrices are simulated using the R package `hglasso` (Tan et al., 2014). The star network and its corresponding data matrix are simulated using the R package `huge` (Zhao et al., 2012). The scale-free network and the inter-hub network are simulated using the R package `igraph` (Csárdi et al., 2024). Additionally, the covariance matrices of the scale-free network, the inter-hub network, and their corresponding data matrices are generated following the procedures outlined in the `hglasso` and `huge` packages, respectively. For more details about the super-hub networks, see (Tan et al., 2014).

The graphical models used in the simulation examples are illustrated in Supplementary Figs S1, S2, S3, S4, and S5. In each Fig, the number of nodes p is equal to 500. The hub nodes are represented by larger solid nodes, while non-hub nodes are depicted as small circles.

1.2 Simulation examples

The following pages include high-resolution summary Figs of the simulation results. The methods are illustrated with box plots, and the model/hub selection criteria are reported in parentheses. We consider two penalization methods here: hub graphical lasso (hglasso) (Tan et al., 2014), and Sparse PArtial Correlation Estimation (`space`) (Peng et al., 2009). hglasso and `space` methods are implemented in the R-packages `hglasso` and `space`, respectively. The methods, along with their corresponding model selection criteria and hub detection procedures, are listed below:

- hglasso and Bayesian Information Criterion type quantity (shortly BIC). We denote this procedure with `hglasso (BIC)`.
- hglasso and MDSD. We denote this procedure with `hglasso (MDSD)`.
- `space` and Bayesian Information Criterion type quantity. Although we refer to this criterion as BIC, it is important to note that it differs from the BIC used by Tan et al. (2014). We denote this procedure with `space (BIC)`.
- `space` and MDSD. We denote this procedure with `space (MDSD)`.

In addition to hglasso and `space`, we employ two quick approximation methods for GGMs that utilize the lossy screening of the sample correlation matrix. These procedures prove particularly useful when examining large-scale co-expression networks (Zhao et al., 2012). These hub detection procedures are listed below:

- The Firouzi and Hero (Hero and Rajaratnam, 2012; Firouzi and Hero, 2013) hub screening method. Here the hub screening is accomplished by thresholding the sample correlation matrix using the threshold value proposed by Firouzi and Hero (2013). We denote this procedure with `cor (FH)`.

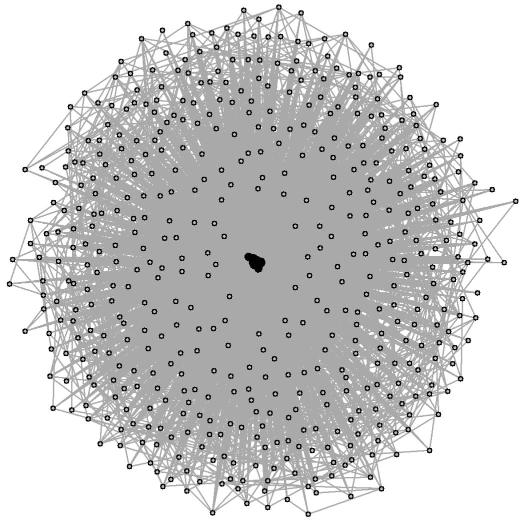

Fig S1. The hub network.

- lossy screening combined with MDSD. We denote this procedure with `cor (MDSD)`.

In each example, we set $\gamma = 3$ while using MDSD.

Estimated values of binary classification metrics are calculated from 100 simulation replications for each example. The sample size and the number of parameters, denoted as (n, p), are either $(100, 500)$ or $(500, 1500)$. The number of parameters (p) is represented in a distinct panel on the right-hand side of the plot. Different methods are distinguished using different colors. The simulation model is reported at the top of each Fig.

The results of the hglasso procedure are summarized in Supplementary Figs S6, S7, S8, S9, and S10. When using hglasso, the models selected either by using BIC or the hubs detected with MDSD depend on the same set of tuning parameter values.

We group the FH screening method and the lossy screening rule, which utilizes MDSD, together, even though the corresponding data-driven networks do not depend on the same set of tuning parameters. Results of these procedures are summarized in Supplementary Figs S11, S12, S13, S14, and S15.

`space` has two tuning parameters, λ_1 and λ_2. We used a grid with 10 values, evenly spaced between 0.1 and 2 for λ_1, and we fixed $\lambda_2 = 0$, meaning that lasso regression is used in the

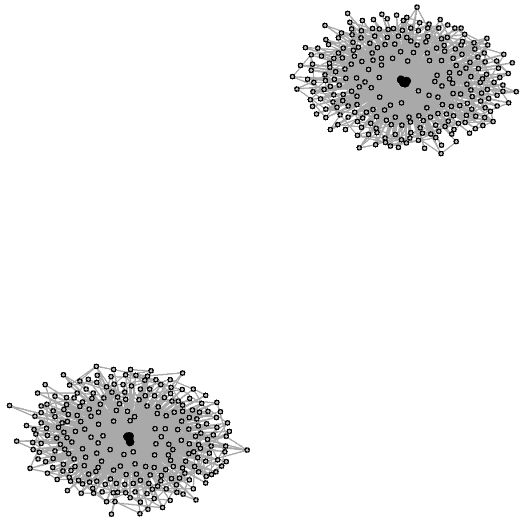

Fig S2. The two-component hub network.

joint sparse regression model. We set the weight parameter as 2, meaning that the estimated degree of each variable is used for weights in the joint sparse regression model. Due to the high computational demand of the R implementation of the `space`, which increases substantially as the number of variables p increases, we limited p to 500 in these simulation examples. In addition, `space` does not provide any tools to determine which nodes are hub nodes, unlike hglasso. Thus, after the network model is selected using BIC, we rank the nodes by their degree and select the top h_n nodes with the highest degree as hub nodes. Here, h_n is equal to the true number of network hubs, as determined from the true graphical model. While using `space`, the models selected either using BIC or the hubs detected with MDSD depend on the same set of tuning parameter values. The results associated with the `space` procedure are summarized in Supplementary Figs S16, S17, S18, S19, and S20.

2 Cut-off parameter γ vs. False Discovery Rate

The estimator used for data-driven network construction substantially affects FDR control. In this example, we use the graphical lasso (glasso) estimator to compute data-driven networks when

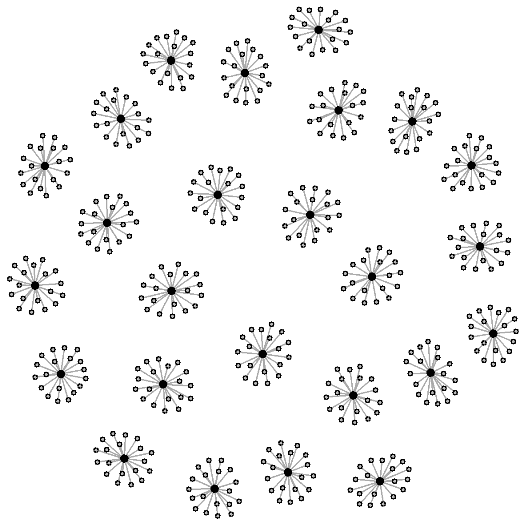

Fig S3. The star network.

the true graphical models used to generate samples from a multivariate Gaussian distribution are the same as those considered in our simulation examples. We set the number of variables as 500 ($p = 500$), and the sample sizes as $n = 100, 300$, and 510. We computed the data-driven network estimate once with each sample size and examined how many true and false hub nodes are detected using MDSD. In particular, we examine how the FDR changes when the values of the cut-off parameters γ are altered. The results are reported in Supplementary Figs S21 - S23.

These examples show that our hub selection procedure controls the FDR of hub node detection quite efficiently across a wide range of cut-off values γ. The FDR values are low and do not change with a wide range of γ values, which means that the procedure is robust to the change in cut-off value. Moreover, when data-driven models whose empirical degree distribution does not resemble a power-law distribution are ignored during MDSD computation, the FDR is even lower.

However, this is only the case when there are clear hub nodes in the network and when the graphical model estimator is adequate for estimating the network. From Supplementary Figs S21 - S23, we can see that the FDR values are extremely high when the true graphical model is the scale-free (Barab'asi-Albert) network considered in our simulation examples. Even when the sample size increases, the estimated FDR does not decrease. This clearly indicates that glasso is not an adequate estimator for this graphical model.

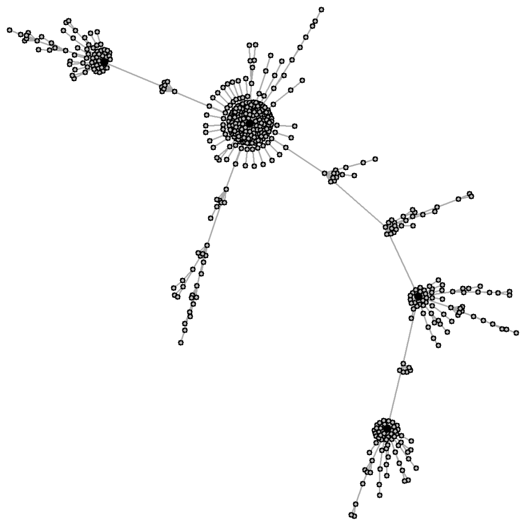

Fig S4. The scale-free (Barabási–Albert) network.

From our previous simulation examples, we see that the averaged FDR values are much lower when `space` method is used to estimate the data-driven network in the scale-free case. When we use `space` method instead of glasso estimator to construct the data-driven network, we again see that our procedure controls the FDR very efficiently across a wide range of cut-off values γ (see Supplementary Figs S24 - S26).

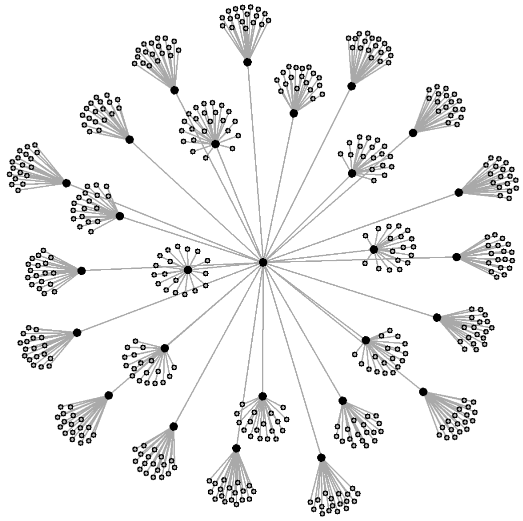

Fig S5. The inter-hub network.

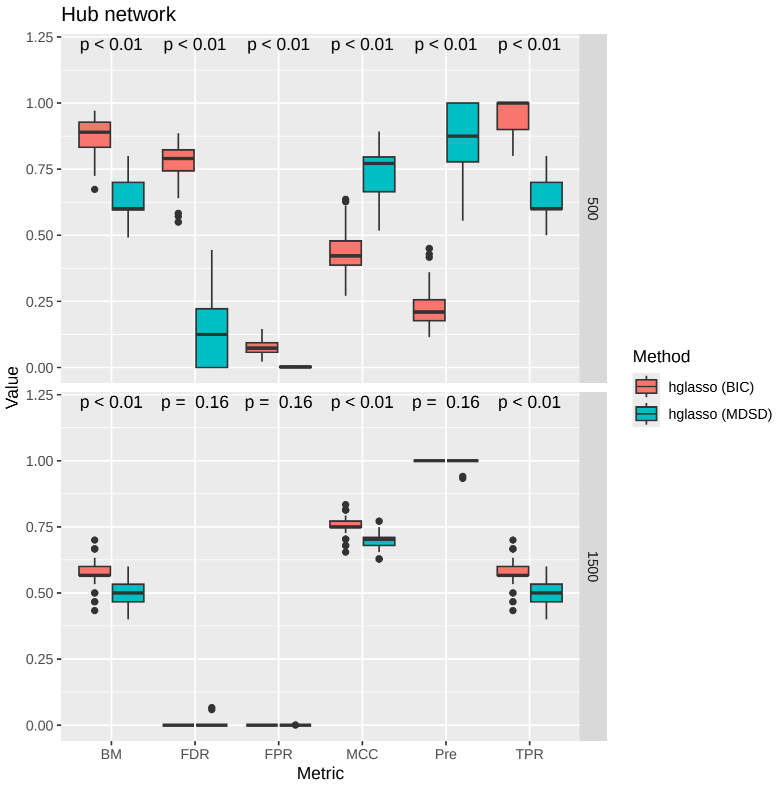

Fig S6. Averaged values of binary classification metrics, estimated from 100 simulation replications. Here hglasso is used to detect hubs with either BIC or MDSD. Hub detection methods are distinguished using different colors and grouped boxplots. The graphical model considered here is the Hub-network. Significance of the average difference between two methods is indicated with a $p-$value (Mann-Whitney U test).

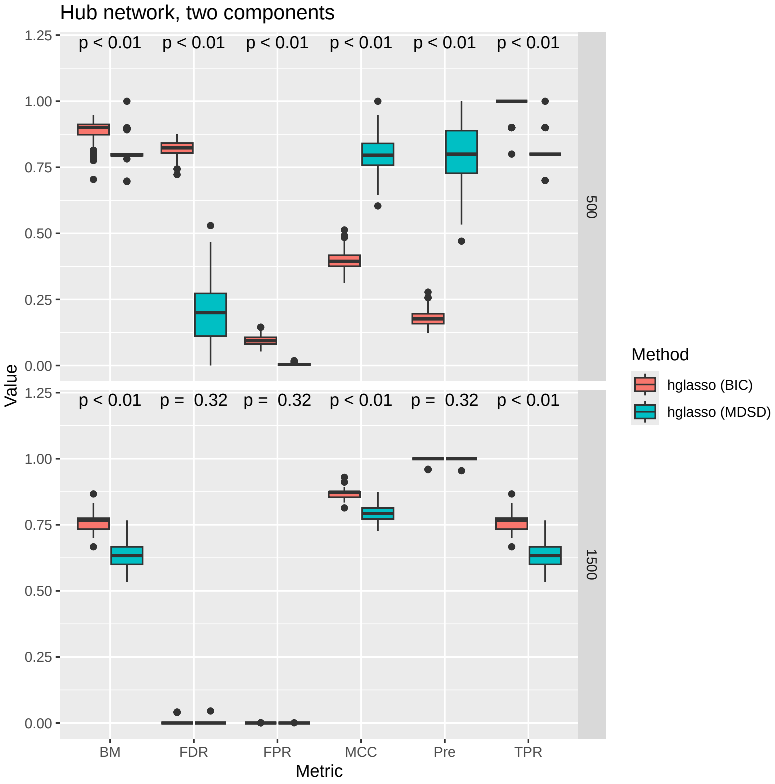

Fig S7. Averaged values of binary classification metrics, estimated from 100 simulation replications. Here hglasso is used to detect hubs with either BIC or MDSD. Hub detection methods are distinguished using different colors and grouped boxplots. The graphical model considered here is the Two component hub-network. Significance of the average difference between two methods is indicated with a $p-$value (Mann-Whitney U test).

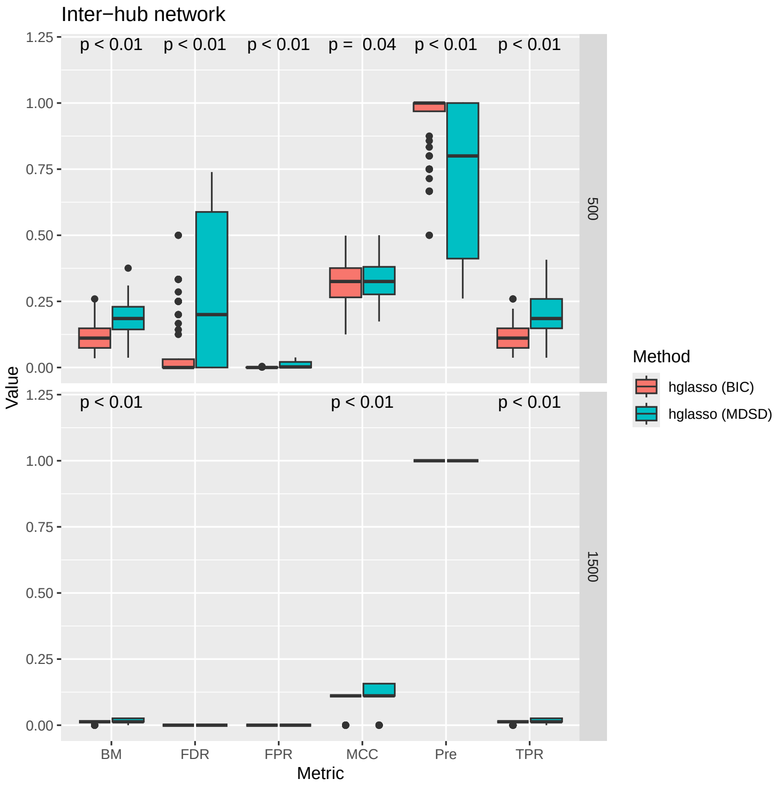

Fig S8. Averaged values of binary classification metrics, estimated from 100 simulation replications. Here hglasso is used to detect hubs with either BIC or MDSD. Hub detection methods are distinguished using different colors and grouped boxplots. The graphical model considered here is the Inter-hub network. Significance of the average difference between two methods is indicated with a $p-$value (Mann-Whitney U test).

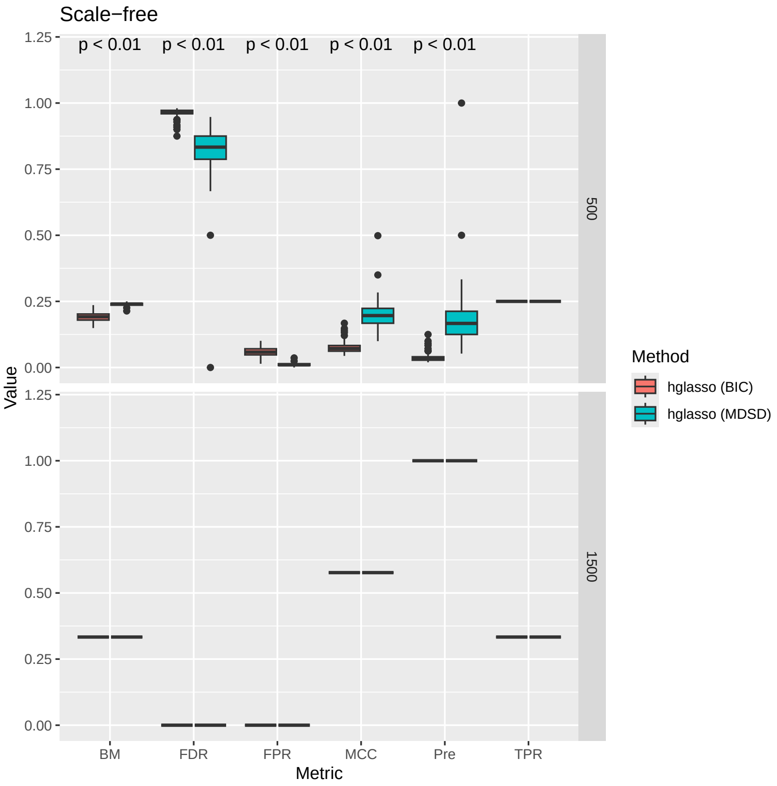

Fig S9. Averaged values of binary classification metrics, estimated from 100 simulation replications. Here hglasso is used to detect hubs with either BIC or MDSD. Hub detection methods are distinguished using different colors and grouped boxplots. The graphical model considered here is the Scale-free network. Significance of the average difference between two methods is indicated with a $p-$value (Mann-Whitney U test).

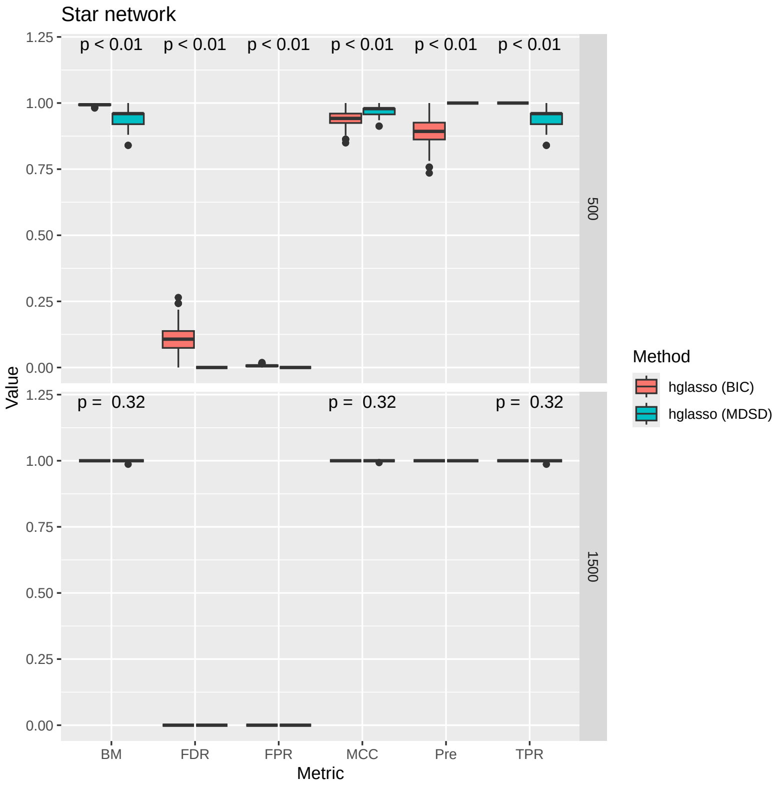

Fig S10. Averaged values of binary classification metrics, estimated from 100 simulation replications. Here hglasso is used to detect hubs with either BIC or MDSD. Hub detection methods are distinguished using different colors and grouped boxplots. The graphical model considered here is the Star network. Significance of the average difference between two methods is indicated with a $p-$value (Mann-Whitney U test).

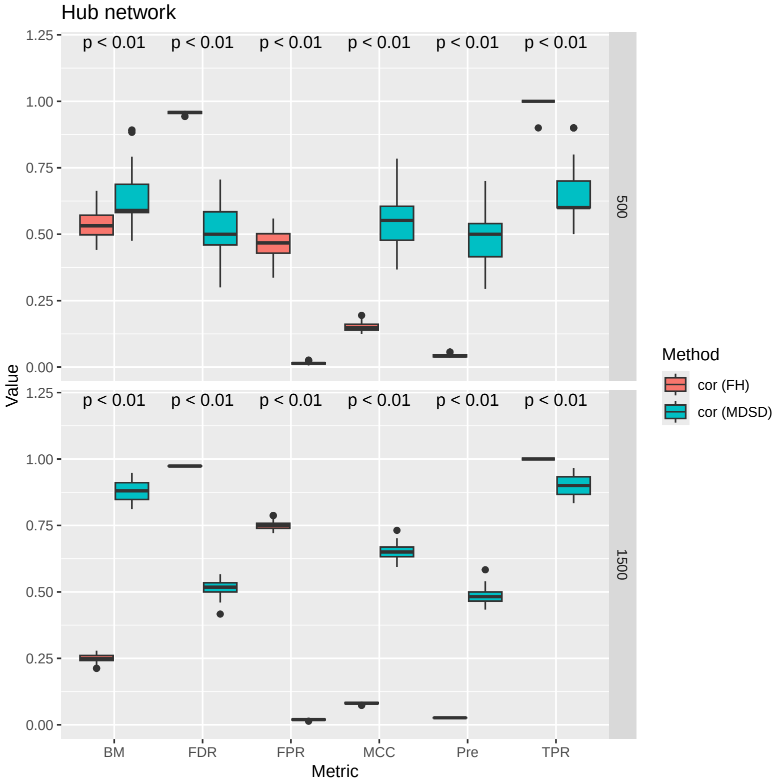

Fig S11. Averaged values of binary classification metrics, estimated from 100 simulation replications. Here lossy screening with MDSD and FH hub screening method are used to detect hubs. Hub detection procedures are distinguished using different colors and grouped boxplots. The graphical model considered here is the Hub-network. Significance of the average difference between two methods is indicated with a $p-$value (Mann-Whitney U test).

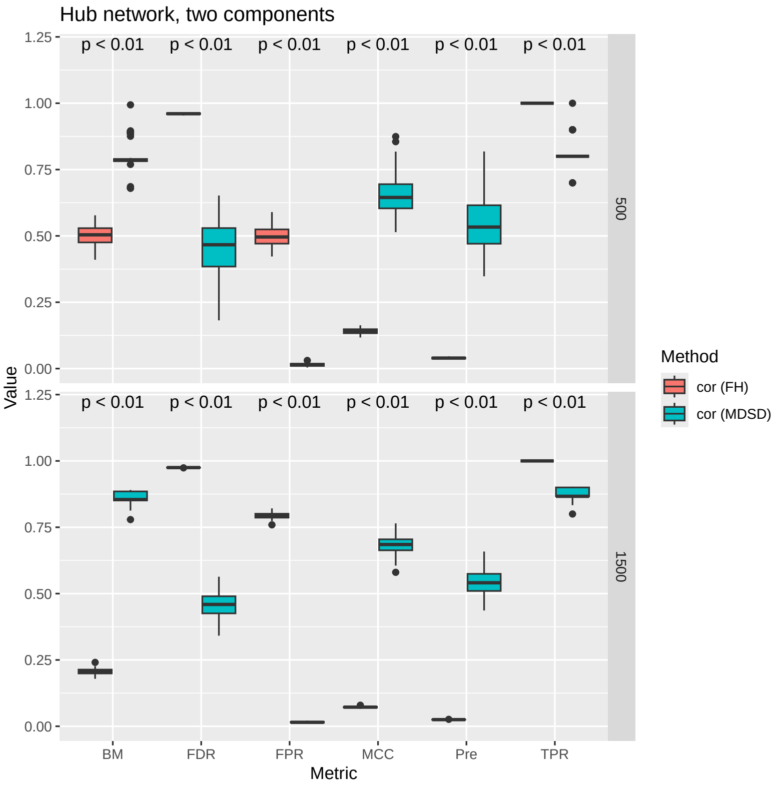

Fig S12. Averaged values of binary classification metrics, estimated from 100 simulation replications. Here lossy screening with MDSD and FH hub screening method are used to detect hubs. Hub detection procedures are distinguished using different colors and grouped boxplots. The graphical model considered here is the Two component hub-network. Significance of the average difference between two methods is indicated with a $p-$value (Mann-Whitney U test).

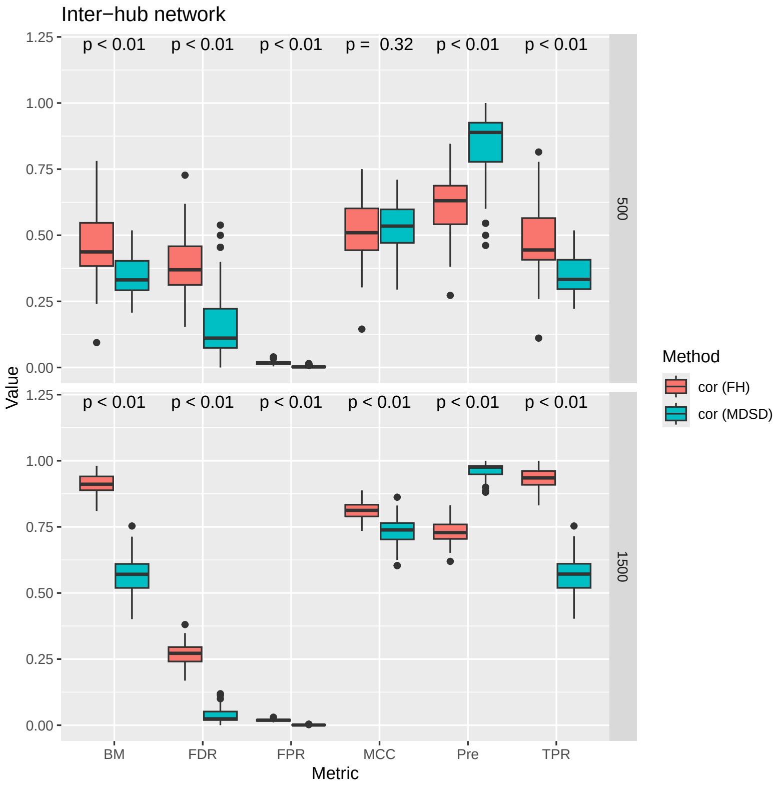

Fig S13. Averaged values of binary classification metrics, estimated from 100 simulation replications. Here lossy screening with MDSD and FH hub screening method are used to detect hubs. Hub detection procedures are distinguished using different colors and grouped boxplots. The graphical model considered here is the Inter-hub network. Significance of the average difference between two methods is indicated with a $p-$value (Mann-Whitney U test).

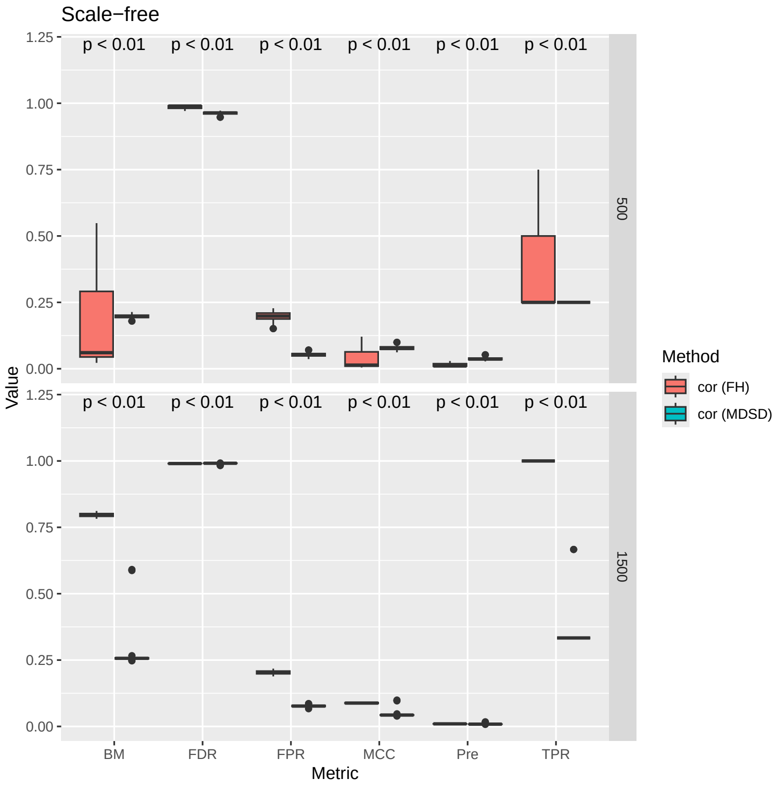

Fig S14. Averaged values of binary classification metrics, estimated from 100 simulation replications. Here lossy screening with MDSD and FH hub screening method are used to detect hubs. Hub detection procedures are distinguished using different colors and grouped boxplots. The graphical model considered here is the Scale-free network. Significance of the average difference between two methods is indicated with a $p-$value (Mann-Whitney U test).

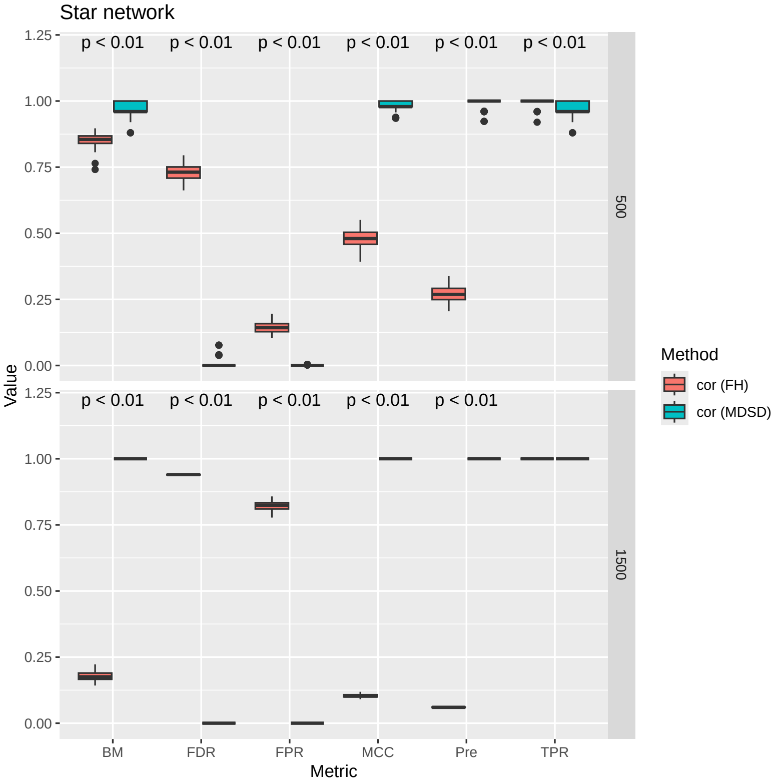

Fig S15. Averaged values of binary classification metrics, estimated from 100 simulation replications. Here lossy screening with MDSD and FH hub screening method are used to detect hubs. Hub detection procedures are distinguished using different colors and grouped boxplots. The graphical model considered here is the Star network. Significance of the average difference between two methods is indicated with a $p-$value (Mann-Whitney U test).

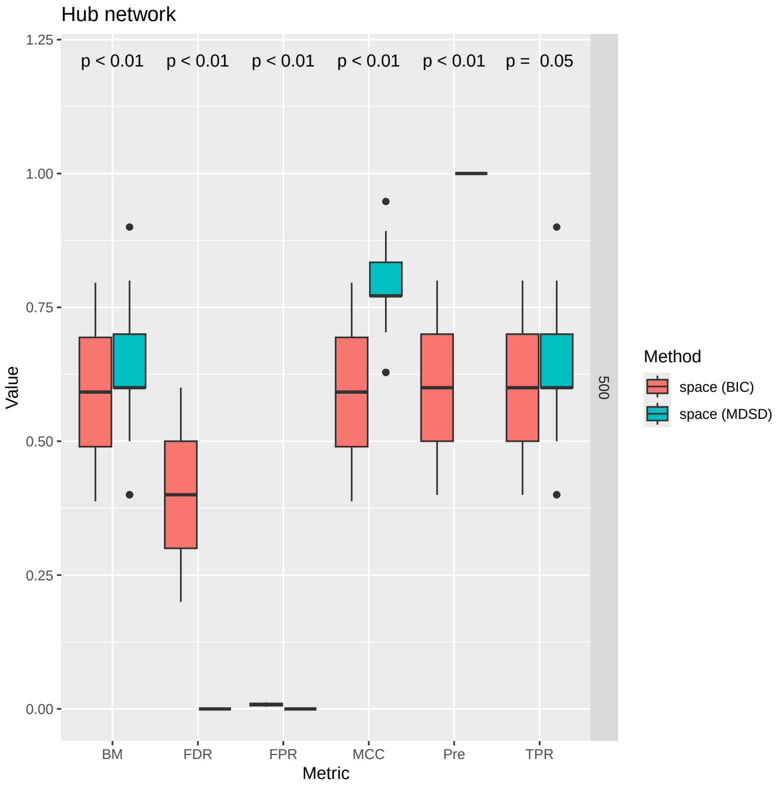

Fig S16. Averaged values of binary classification metrics, estimated from 100 simulation replications. Here `space` is used to detect hubs with either BIC or MDSD. Hub detection methods are distinguished using different colors and grouped boxplots. The graphical model considered here is the Hub-network. Significance of the average difference between two methods is indicated with a $p-$value (Mann-Whitney U test).

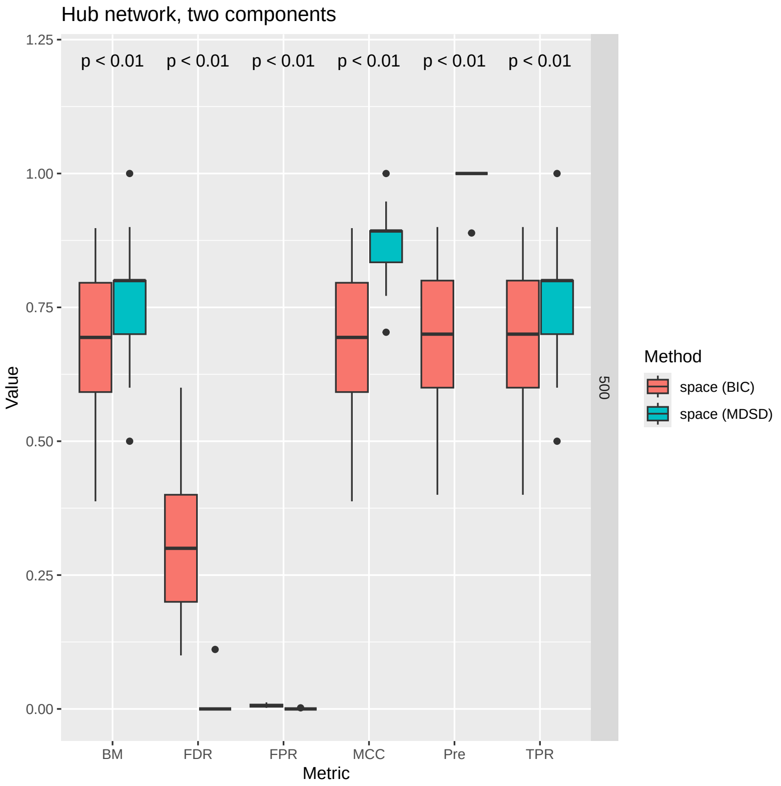

Fig S17. Averaged values of binary classification metrics, estimated from 100 simulation replications. Here `space` is used to detect hubs with either BIC or MDSD. Hub detection methods are distinguished using different colors and grouped boxplots. The graphical model considered here is the Two component hub-network. Significance of the average difference between two methods is indicated with a $p-$value (Mann-Whitney U test).

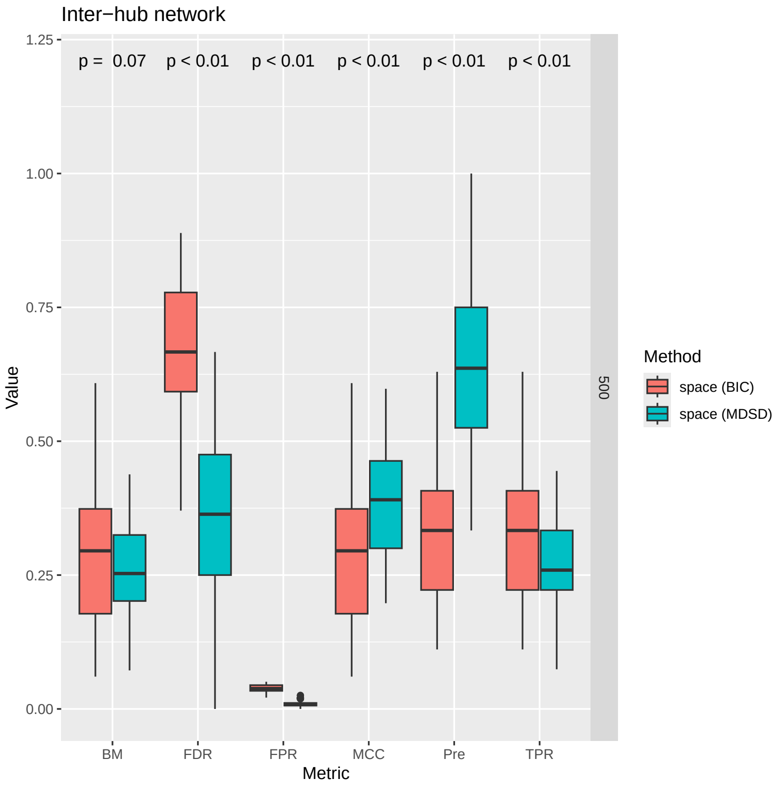

Fig S18. Averaged values of binary classification metrics, estimated from 100 simulation replications. Here `space` is used to detect hubs with either BIC or MDSD. Hub detection methods are distinguished using different colors and grouped boxplots. The graphical model considered here is the Inter-hub network. Significance of the average difference between two methods is indicated with a $p-$value (Mann-Whitney U test).

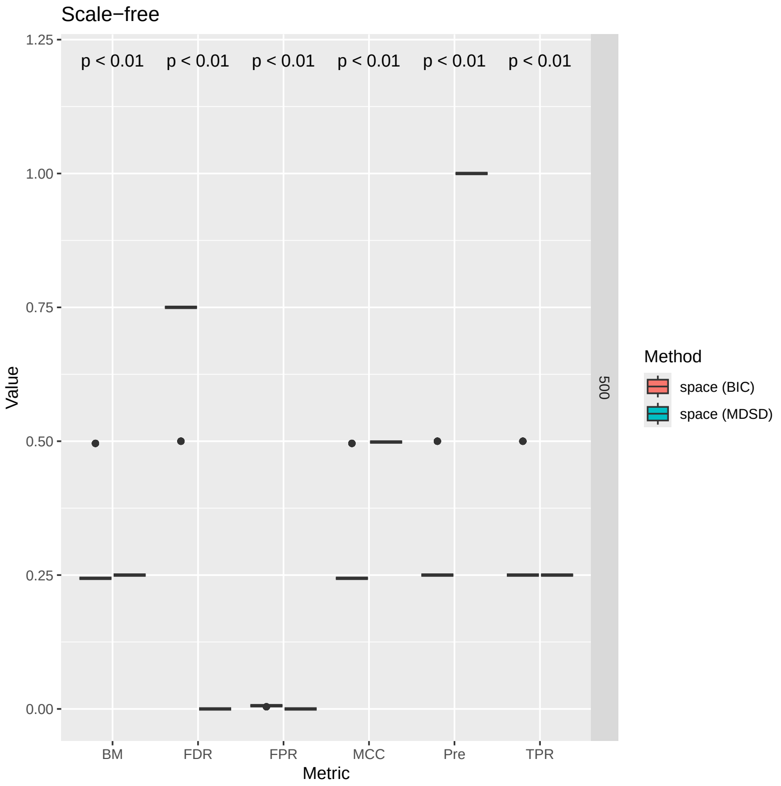

Fig S19. Averaged values of binary classification metrics, estimated from 100 simulation replications. Here `space` is used to detect hubs with either BIC or MDSD. Hub detection methods are distinguished using different colors and grouped boxplots. The graphical model considered here is the Scale-free network. Significance of the average difference between two methods is indicated with a $p-$value (Mann-Whitney U test).

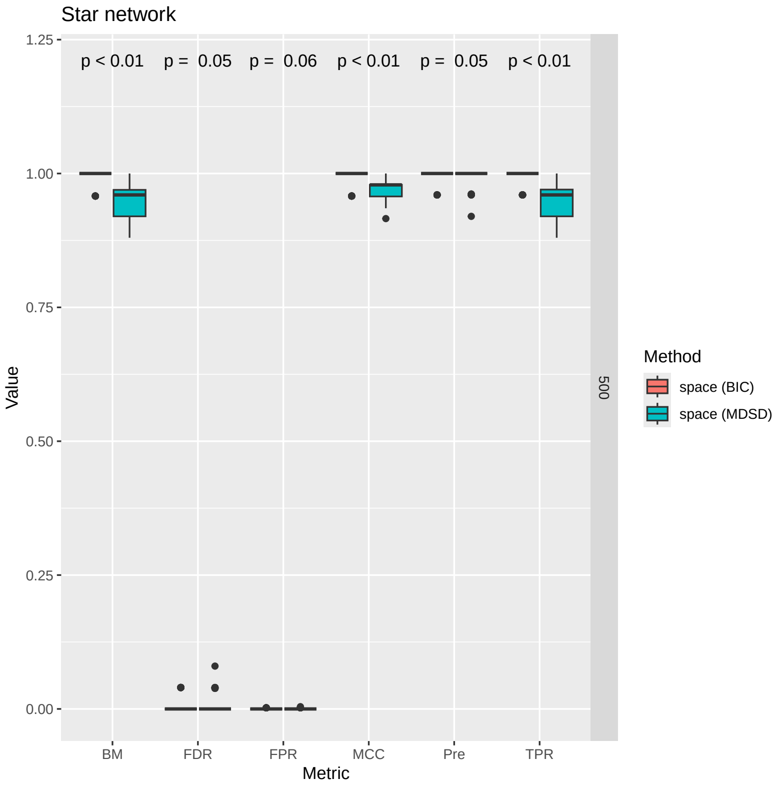

Fig S20. Averaged values of binary classification metrics, estimated from 100 simulation replications. Here `space` is used to detect hubs with either BIC or MDSD. Hub detection methods are distinguished using different colors and grouped boxplots. The graphical model considered here is the Star network. Significance of the average difference between two methods is indicated with a $p-$value (Mann-Whitney U test).

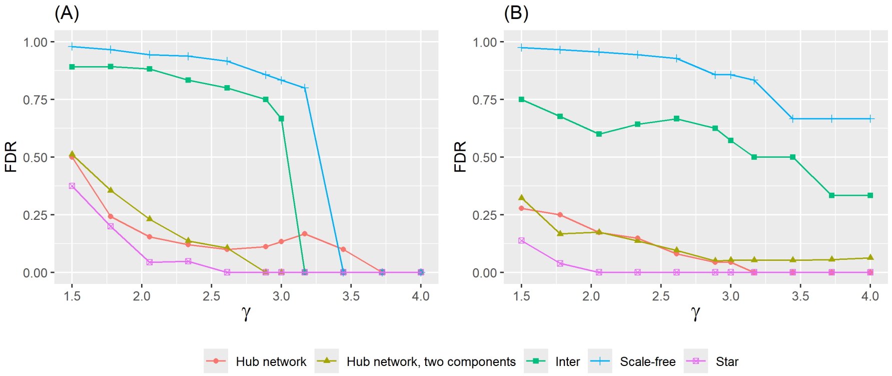

Fig S21. The estimated FDR values as a function of γ when graphical lasso (glasso) estimator is used, the sample size $n = 100$. Different simulated network models are presented using different colors and symbols. (A) When the whole solution path is used to compute MDSD. (B) When ignoring models with estimated degree distribution skewness smaller than 0.5 from MDSD computations.

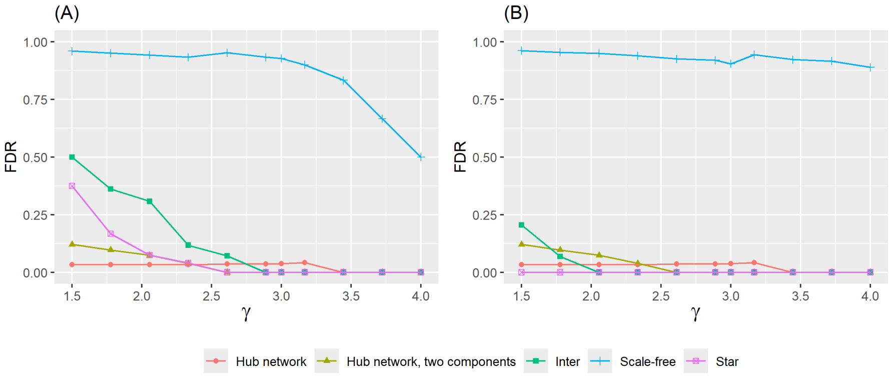

Fig S22. The estimated FDR values as a function of γ when graphical lasso (glasso) estimator is used, the sample size $n = 300$. Different simulated network models are presented using different colors and symbols. (A) When the whole solution path is used to compute MDSD. (B) When ignoring models with estimated degree distribution skewness smaller than 0.5 from MDSD computations.

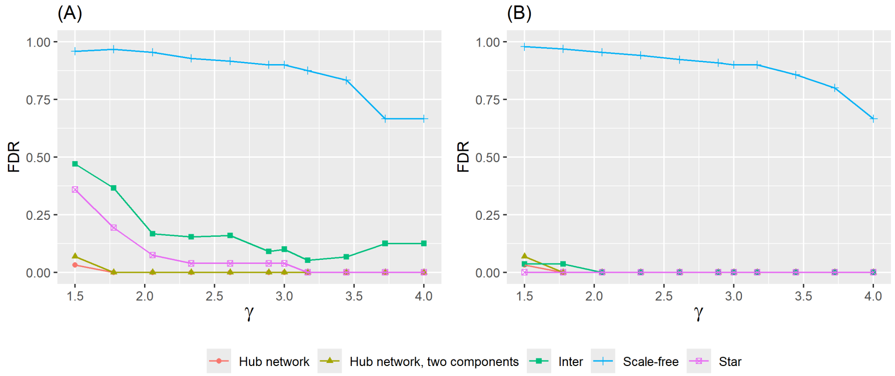

Fig S23. The estimated FDR values as a function of γ when graphical lasso (glasso) estimator is used, the sample size $n = 510$. Different simulated network models are presented using different colors and symbols. (A) When the whole solution path is used to compute MDSD. (B) When ignoring models with estimated degree distribution skewness smaller than 0.5 from MDSD computations.

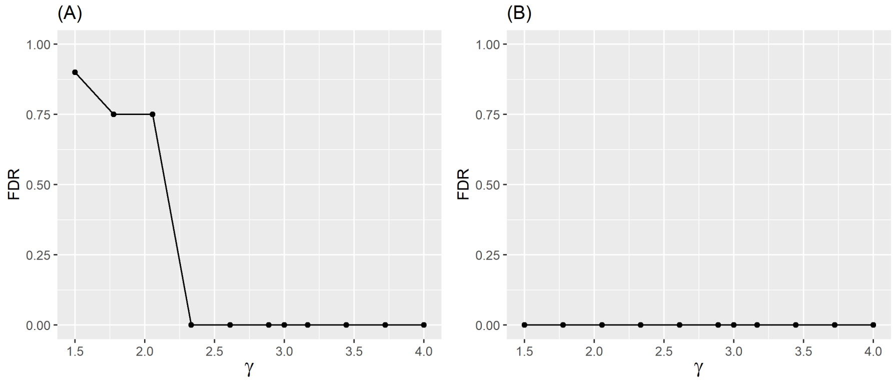

Fig S24. The estimated FDR values as a function of γ when `space` method is used, the sample size $n = 100$. (A) When the whole solution path is used to compute MDSD. (B) When ignoring models with estimated degree distribution skewness smaller than 0.5 from MDSD computations. The graphical model considered here is the Scale-free network.

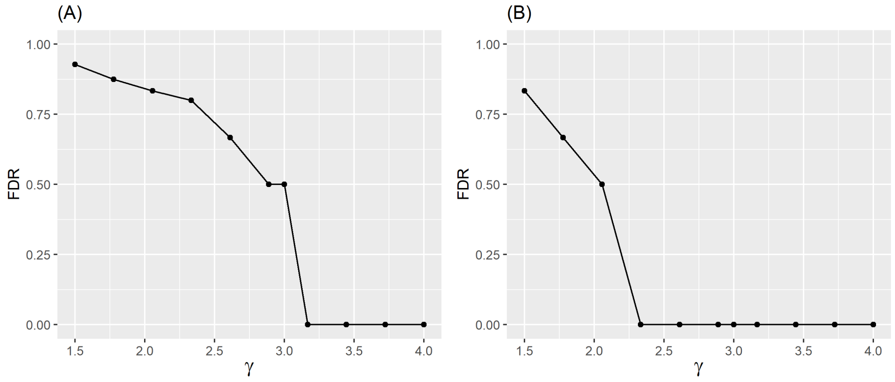

Fig S25. The estimated FDR values as a function of γ when `space` method is used, the sample size $n = 300$. (A) When the whole solution path is used to compute MDSD. (B) When ignoring models with estimated degree distribution skewness smaller than 0.5 from MDSD computations. The graphical model considered here is the Scale-free network.

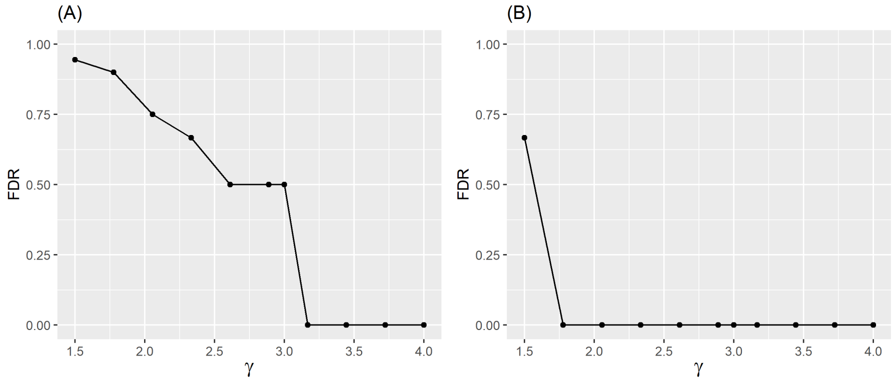

Fig S26. The estimated FDR values as a function of γ when `space` method is used, the sample size $n = 510$. (A) When the whole solution path is used to compute MDSD. (B) When ignoring models with estimated degree distribution skewness smaller than 0.5 from MDSD computations. The graphical model considered here is the Scale-free network.

References

Csárdi, G., Nepusz, T., Traag, V., Horvát, S., Zanini, F., Noom, D., and Müller, K. (2024). *igraph: Network Analysis and Visualization in R*. https://doi.org/10.5281/zenodo.7682609.

Firouzi, H. and Hero, A. O. (2013). Local hub screening in sparse correlation graphs. In Ville, D. V. D., Goyal, V. K., and Papadakis, M., editors, *Wavelets and Sparsity XV*, volume 8858, page 88581H. International Society for Optics and Photonics, SPIE. https://doi.org/10.1117/12.2024361.

Hero, A. and Rajaratnam, B. (2012). Hub discovery in partial correlation graphs. *IEEE Transactions on Information Theory*, 58:6064 – 6078. https://doi.org/10.1109/TIT.2012.2200825.

Peng, J., Wang, P., Zhou, N., and Zhu, J. (2009). Partial correlation estimation by joint sparse regression models. *Journal of the American Statistical Association*, 104:735–746. https://doi.org/10.1198/jasa.2009.0126.

Tan, K. M., London, P., Mohan, K., Lee, S.-I., Fazel, M., and Witten, D. (2014). Learning graphical models with hubs. *Journal of Machine Learning Research*, 15:3297–3331. http://jmlr.org/papers/v15/tan14b.html.

Zhao, T., Liu, H., Roeder, K., Lafferty, J., and Wasserman, L. (2012). The huge package for high-dimensional undirected graph estimation in R. *Journal of Machine Learning Research*, 13:1059–1062. http://jmlr.org/papers/v13/zhao12a.html.
